# Prioritizing water availability study settings to address geogenic contaminants and related societal factors

**DOI:** 10.1007/s10661-024-12362-2

**Published:** 2024-02-24

**Authors:** Melinda L. Erickson, Craig J. Brown, Elizabeth J. Tomaszewski, Joseph D. Ayotte, John K. Böhlke, Douglas B. Kent, Sharon Qi

**Affiliations:** 1 U.S. Geological Survey, 2280 Woodale Drive, Mounds View, MN 55112 USA; 2 U.S. Geological Survey, 101 Pitkin Street, East Hartford, CT 06108 USA; 3 U.S. Geological Survey, 12201 Sunrise Valley Dr, Reston, VA 20192 USA; 4 U.S. Geological Survey, 331 Commerce Way, Pembroke, NH 03275 USA; 5 U.S. Geological Survey, 345 Middlefield Rd, Menlo Park, CA 94025 USA; 6 U.S. Geological Survey, 601 SW 2nd Ave. Suite 1950, Portland, OR 97204 USA

**Keywords:** Geogenic contaminants, Arsenic, Groundwater, Hydrology, Federal research, Environmental justice

## Abstract

Water availability for human and ecological uses depends on both water quantity and water quality. The U.S. Geological Survey (USGS) is developing strategies for prioritizing regional-scale and watershed basin-scale studies of water availability across the nation. Previous USGS ranking processes for basin-scale studies incorporated primarily water *quantity* factors but are now considering additional water *quality* factors. This study presents a ranking based on the potential impacts of geogenic constituents on water quality and consideration of societal factors related to water quality. High-concentration geogenic constituents, including trace elements and radionuclides, are among the most prevalent contaminants limiting water availability in the USA and globally. Geogenic constituents commonly occur in groundwater because of subsurface water–rock interactions, and their distributions are controlled by complex geochemical processes. Geogenic constituent mobility can also be affected by human activities (e.g., mining, energy production, irrigation, and pumping). Societal factors and relations to drinking water sources and water quality information are often overlooked when evaluating research priorities. Sociodemographic characteristics, data gaps resulting from historical data-collection disparities, and infrastructure condition/age are examples of factors to consider regarding environmental justice. This paper presents approaches for ranking and prioritizing potential basin-scale study areas across the contiguous USA by considering a suite of conventional physical and geochemical variables related to geogenic constituents, with and without considering variables related to societal factors. Simultaneous consideration of societal and conventional factors could provide decision makers with more diverse, interdisciplinary tools to increase equity and reduce bias in prioritizing focused research areas and future water availability studies.

**Supplementary Information:**

The online version contains supplementary material available at 10.1007/s10661-024-12362-2.

## Introduction

**Fig. 1:**
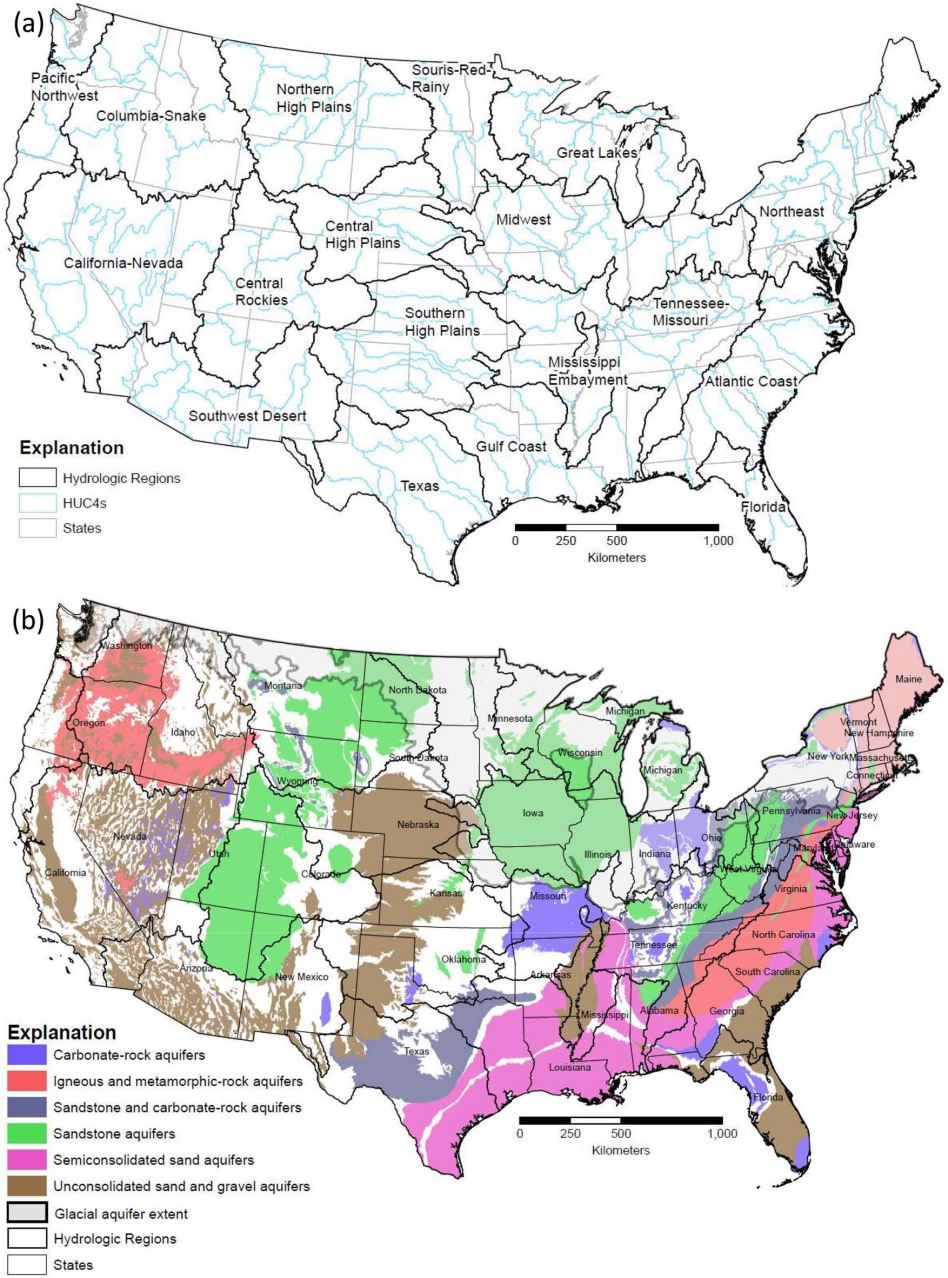
Hydrologic regions (black boundary lines), candidate watershed basins (blue boundary lines), and selected principal aquifers by lithology (shaded areas). **a** Eighteen hydrologic regions and 163 candidate basins (Van Metre et al., 2020); and **b** hydrologic regions and selected principal aquifers by lithology (Miller, 1999)

Sustaining the availability of water for human and ecosystem needs depends on of both water *quantity* and water *quality* (Evenson et al., 2013). The U.S. Geological Survey (USGS) mission includes providing data and interpretive science to understand water resource availability in the USA, including identifying factors that do or may limit water availability (Miller et al., 2020). Current efforts include regional and national integrated water availability assessments in coordination with relatively intensive investigations in selected river basins within the contiguous USA (CONUS), which were selected through a quantitative prioritization scheme (Van Metre et al., 2020). The framework for the river basin prioritization scheme consisted of 163 candidate basins that were level-4 hydrologic units (HUC04) (U.S. Geological Survey, 2023), modified in some cases to combine smaller basins, and distributed among 18 hydrologic regions (Fig. 1). Candidate basins were ranked nationally and within each region, primarily on the basis of anthropogenic stressors of surface water resource quantity (Van Metre et al., 2020). The Van Metre et al. (2020) basin prioritization scheme did not consider water availability factors such as groundwater quality or societal factors related to environmental justice (EJ). Groundwater supplies drinking water to approximately 130 million people, about one-third of the US population, with about 40 million people deriving drinking water solely from self-supplied domestic well water (DeSimone et al., 2015). The current study presents a modified approach for quantitative prioritization of study basins, which incorporates groundwater quality and societal aspects of water availability.

Our study focus is groundwater containing elevated concentrations of potentially harmful dissolved geogenic constituents, which are defined as chemicals or isotopes that have geologic or atmospheric sources (although they can also have other sources, as described in the supplemental information and in Erickson et al. (2024)). Geogenic trace metals, other trace elements, and radionuclides have been linked to increased cancer risk (Agency for Toxic Substances and Disease Registry, 1999; Krajewski et al., 2021; Mendez et al., 2017) and non-cancer adverse human health outcomes (Agency for Toxic Substances and Disease Registry, 1999; Larvie et al., 2022; Naujokas et al., 2013; Wasserman et al., 2016). Because of their widespread occurrence in geologic and other materials, geogenic constituents are among the most prevalent contaminants found in drinking source water, thus limiting drinking water availability in the USA and globally (Smedley & Kinniburgh, 2002; Welch et al., 2000). Multiple geogenic constituents are common and widely distributed at “high” concentrations (exceeding a current regulatory threshold such as the U.S. Environmental Protection Agency (EPA) Maximum Contaminant Level (MCL), or exceeding other human health benchmark values) and even more common at elevated concentrations (defined herein as exceeding one-half of threshold values) in aquifers underlying the hydrologic regions across the USA (Table 1, Fig. 1) (Norman et al., 2018; State of California, 2000; U.S. Environmental Protection Agency, 2018). Drinking water thresholds have been developed for many geogenic constituents, but it is also important to understand the wider distribution of sub-regulatory concentrations (Table 1) because (1) regulatory thresholds can change, (2) regulatory thresholds are based on economic factors in balance with human health effect (U.S. Environmental Protection Agency, 2023), (3) effects of mixtures are not well known, (4) drinking water thresholds are not enforceable for private domestic wells (U.S. Environmental Protection Agency, 2022), (5) concentrations can change because of human activities, and (6) studying a wide range of occurrences can improve understanding of sources and environmental controls. Arsenic is used as a representative geogenic constituent in this study because it was the only geogenic constituent modeled at the CONUS scale as of 2023.

**Table 1:**
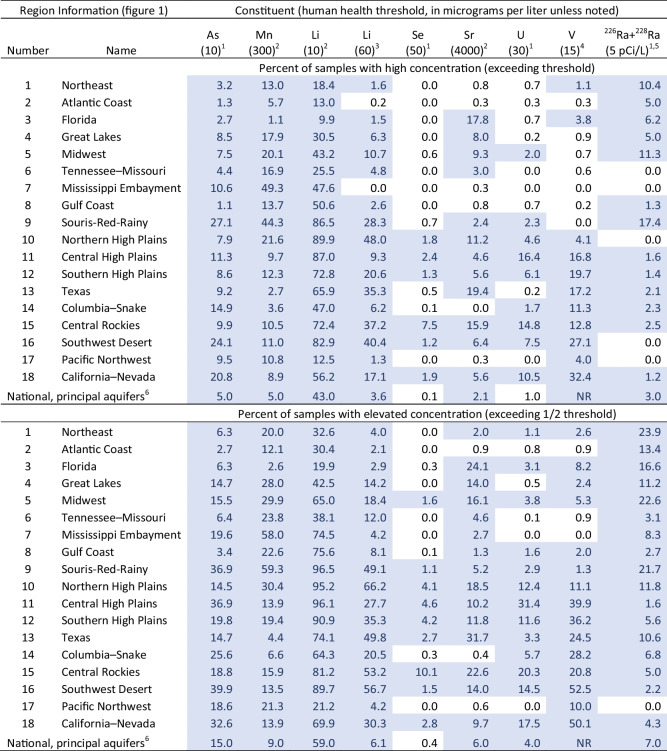
Regional and national exceedances of human health drinking water thresholds for selected constituents in groundwater samples from wells in the USA

*As*, arsenic; *Mn*, manganese; *Li*, lithium; *Se*, selenium; *Sr*, strontium; *V*, vanadium; *Ra*, radium; *High*, concentration exceeding a human health threshold; *Elevated*, concentration exceeding 1/2 human health threshold; *NR*, not reported; blue shading indicates > 1% of samples have elevated or high concentrations; regional data extracted from the National Water Information System (U.S. Geological Survey, 2019). Some regions may be represented by relatively few data; see method described in Supplemental Information and regional sample counts in Table S3

^1^*MCL*, Environmental Protection Agency maximum contaminant level (U.S. Environmental Protection Agency, 2018)

^2^*HBSL*, health-based screening level (Norman et al., 2018)

^3^Assumes that 100% of exposure to lithium comes from drinking water (Lindsey et al., 2021)

^4^*AL*, State of California action level (State of California, 2000)

^5^*pCi/L*, picocuries per liter

^6^Groundwater source water serving public water systems. Data from Belitz et al. (2022)

Geogenic constituents generally occur more widely at higher concentrations in groundwater compared to surface water because of the interaction of subsurface water with soils and aquifer material. Constituent concentrations in upper-crustal materials are commonly sufficient that dissolution of small fractions during water–rock interaction can cause an exceedance of concentration thresholds for intended water use (Table S1). Concentrations of many geogenic constituents increase with groundwater age and flowpath length but also vary with aquifer lithology and hydrogeochemical conditions including pH and redox (DeSimone & Ransom, 2021; Erickson et al. 2021a, 2021b; Knierim et al., 2022; Lindsey et al., 2021; Stackelberg et al., 2021).

Geogenic constituents can be grouped loosely by mobilization processes and sources (Table 2, Figure S1) to include (A) oxic waters, (B) acidic waters, (C) reducing waters, (D) radionuclides, (E) saline (high ionic strength) waters, and (F) pipe corrosion that can be exacerbated by corrosive source water chemistry, atmospheric deposition, and mixtures (Table 2, Figure S1). Groupings A–C (Table 2) are mobilized primarily by varying pH and redox, as described in detail in Figure S1. Aqueous radionuclides such as U (uranium), Rn (radon), Ra (radium), and Th (thorium) commonly result from the natural weathering of aquifer minerals, as described in Figure S1 (element abbreviations defined in Table S1). Geogenic constituents are commonly associated with saline, high ionic strength waters, such as brines and brackish groundwater, wastewater from oil and gas production, or salt-water intrusion (Stanton et al., 2017). If source water is corrosive, pipe corrosion can release metals into tap water (Jurgens et al., 2019). Atmospheric deposition can be a geogenic source of constituents such as Hg (mercury), NO_3_^−^ (nitrate), and ClO_4_^−^ (perchlorate). Concentrations of geogenic constituents can also be enriched by ion exchange and evaporative concentration (Table 2; Figure S1). NO_3_^−^ and ClO_4_^−^, for example, can be enriched to high concentrations in the unsaturated zone of semi-arid to arid environments through evapoconcentration (Jackson et al., 2015; Rajagopalan et al., 2006). Mixtures of geogenic constituents, even at concentrations below thresholds, can result in more serious health effects than individual constituents (Wang & Fowler, 2008). Many anthropogenic activities can exacerbate geogenic constituent mobilization, either directly (e.g., application of fertilizers on the land surface or releases resulting from resource extraction) or indirectly (e.g., alteration of geochemical or hydrologic conditions, which in turn affects constituent solubility or mobility). For example, irrigation and artificial recharge can flush accumulated unsaturated zone salts into groundwater. Climate can also affect the occurrence and distribution of geogenic constituents in groundwater (Ayotte et al., 2011b; Erickson et al., 2023; Lombard et al. 2021b; Tesoriero et al., 2023).

**Table 2:** Selected geogenic constituents grouped by mobilization processes, sources, and occurrence. Processes are depicted and described in more detail in supplemental Figure S1. Note that some constituents can be included in more than one grouping, and concentrations can be enriched by ion exchange and evaporative concentration

Figure S1 panel	Categories	Selected constituents	Conditions or associations
A)	Oxic waters	Oxyanions: As(V), Mo(VI), V(V), Cr(VI), Se(VI), Sb(V), U(VI), nitrate, borate	Oxic to suboxic, neutral-alkaline pH waters
B)	Acidic waters	Al, As, Be, Cd, Cr, Cu, Fe, Mn, Ni, Pb, Sb, Tl, Hg	Acid-mine drainage, weathering of sulfide minerals
C)	Reducing waters	As(III), Mn(II), Fe(II), Cr(III), Sb(III), DOC, methane (CH_4_), ammonium (NH_4_^+^)	Suboxic to anoxic waters, organic-rich environments
D)	Radionuclides	U, Th, Ra, Rn, Po	Water–rock interaction, saline waters, variable redox, and pH
E)	Saline waters	Cl, Ba, F, Li, Sr, sulfate (SO_4_^−2^)	Brines, oil and gas development, seawater intrusion
F)	Pipe corrosion	Pb, Cu, V, Zn (associated with plumbing)	Indirect effect of corrosive water source (e.g., pH, DIC, DOC)
A) and B)	Atmospheric	Hg, N, perchlorate	Deposition, evaporative concentration, arid regions, irrigation flushing
All	Constituent mixtures	Multiple geogenic constituents at moderate to high concentrations	Mixtures common nationally; see Table 1

*Al*, aluminum; *As*, arsenic; *Be*, beryllium; *Cd*, cadmium; *Cl*, chloride; *Cr*, chromium; *Cu*, copper; *Fe*, iron; *F*, fluoride; *Hg*, mercury; *Li*; lithium; *Mn*, manganese; *Mo*, molybdenum; *N*, nitrogen; *Ni*, nickel; *Pb*, lead; *Po*, polonium; *Ra*, radium; *Rn*, radon; *Sb*, antimony; *Se*, selenium; *Sr*, strontium; *Tl*, thallium; *U*, uranium; *V*, vanadium; *Zn*, zinc; roman numerals indicate oxidation state; *DIC*, dissolved inorganic carbon; *DOC*, dissolved organic carbon; *NA*, not applicable

Societal factors such as income or race, proximity to pollution sources, and knowledge (or lack of knowledge) about environmental conditions can also be associated with important disparities in water quality and availability related to geogenic constituents. Recent studies highlight drinking water quality inequities related to societal factors including historical economic and racial disparities (Cutter et al., 2003; Nigra et al., 2022; Ravalli et al., 2022; Tanana et al., 2021). Additionally, people with private domestic wells as their drinking water source are more susceptible to exposure to contaminants compared to people served by public water systems because of a lack of required water testing or water treatment (Gibson et al., 2020; Spaur et al., 2021). In some areas, predominantly African American and Latinx communities rely on private domestic wells because of historical barriers to public water system access (Gibson & Pieper, 2017; Purifoy, 2021; Wilson et al., 2008). A recent statistical analysis found that As and radionuclide violations in public water systems were driven primarily by physical factors such as arid climate, but the temporal persistence of violations was driven by societal factors (Scanlon et al., 2022). Nigra et al. (2022) found that high As (arsenic) concentrations are more likely in public water supplies serving lower-income populations, and Ravalli et al. (2022) found relatively higher U, Cr (chromium), Ba (barium), and Se (selenium) concentrations in public water supplies serving Hispanic communities. Certain Native American communities are near historical mining areas, or depend on private domestic well water that has high U and As concentrations (Tanana et al., 2021). Two studies illustrate that As exposure declined for people served by public water systems after the EPA MCL for As was tightened in 2006 (lowered from 50 μg/L to 10 μg/L), whereas people using private domestic wells did not experience the As exposure decline (Nigra et al., 2017; Welch et al., 2018). Testing for As or other contaminants is uncommon in private domestic wells. Household water treatment systems, even when present, are often poorly maintained because of cost and other factors (Flanagan et al., 2016). There are substantial socioeconomic disparities in private domestic well testing and treatment in the USA (Flanagan et al., 2016; Malecki et al., 2017; Yang et al., 2020).

Societal aspects of water availability, including drinking water source and missing water quality information, are often overlooked when evaluating, determining, and ranking the merit and benefit of research, as was the case in Van Metre et al. (2020). To avoid perpetuating these types of historical disparities in water availability research, ranking schemes to prioritize research activities can incorporate societal factors, drinking water source, and data gaps. For example, the State of California now incorporates both physical and societal risk factors (e.g., income, demographics) in calculating drought and water shortage risks (California Department of Water Resources, 2022). This paper presents an approach to quantitatively consider and incorporate both physical–chemical and societal factors in ranking or prioritizing basins for water availability research specific to geogenic constituents. The analysis fills an important gap in understanding of limitations on groundwater availability for human and ecological uses.

## Approach

The general approach for prioritizing study basins based on geogenic constituents and societal factors consisted of (1) Identifying a short list of relevant variables with national geospatial coverage and apportioning the data among the candidate basins and (2) ranking the basins by their relative scores for the selected variables, individually and combined, both nationally and regionally. In order to conduct the prioritization, the variables chosen must be based on information that is available across the CONUS on a sufficiently fine scale to be reliably scaled to HUC04 basin-scale. We explore the prioritization scheme further by comparing results with and without the incorporation of variables associated with societal factors.

Although principal aquifers (Miller, 1999) used as major drinking water sources are not wholly aligned with surface water drainage basins, we adopted the basin-based geographic framework described by Van Metre et al. (2020). The CONUS was divided into 18 hydrologic regions, and candidate basins within each region were the modified HUC04 (median candidate basin area is 46,600 km^2^ (Fig. 1a)). The 18 hydrologic regions represent within-region homogeneity of major hydrologic drivers and processes while maximizing heterogeneity among the regions. The 163 candidate basins provide a consistent framework for the multiple USGS ranking efforts and are a suitable way to break up the principal aquifers into rankable units (Fig. 1, Table S2).

**Fig. 2:**
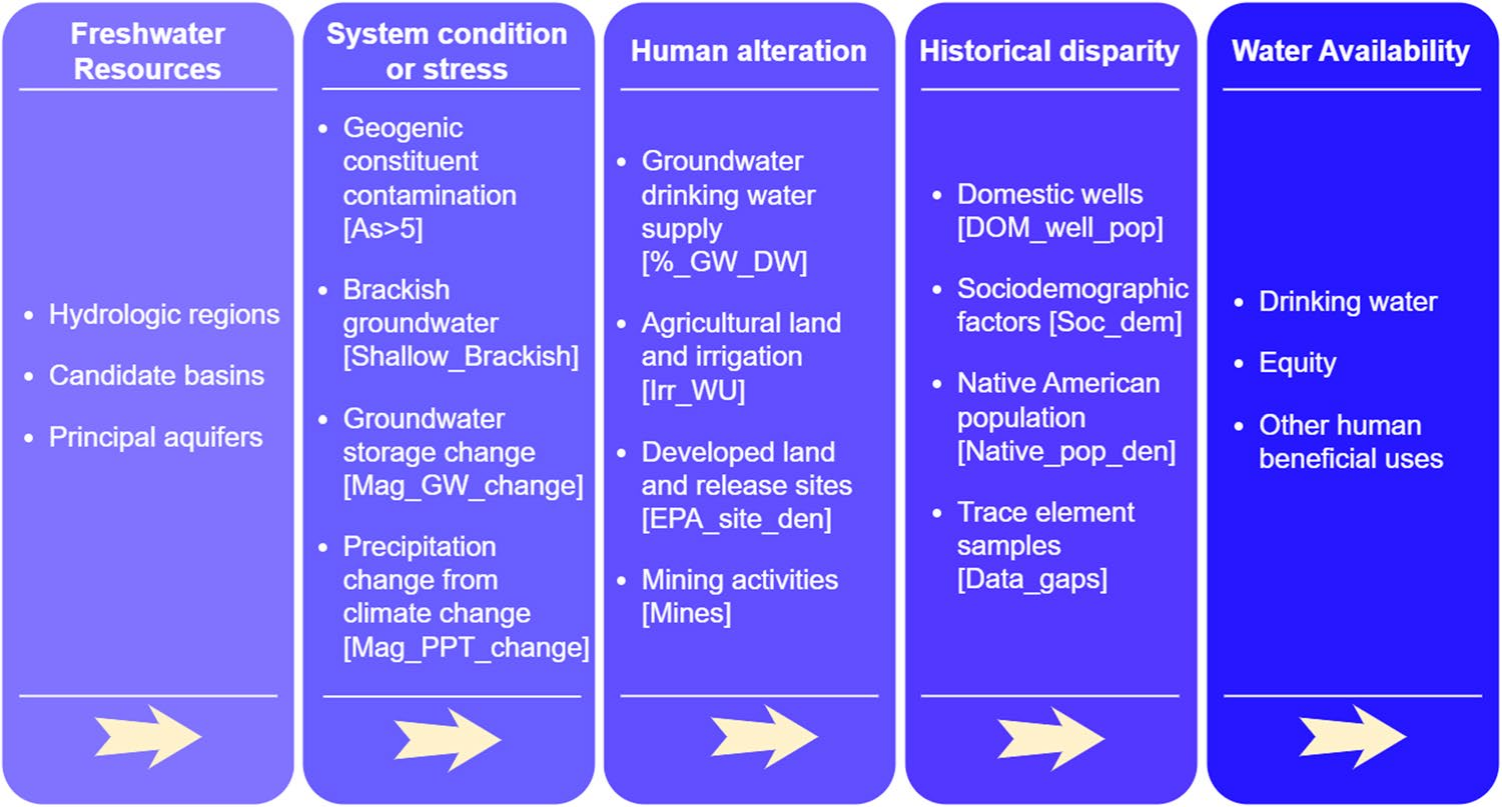
Conceptual model that considers natural conditions, human stressors, and societal factors relevant to understanding and prioritizing geogenic-focused water quality research

We developed a conceptual model that considers several factors relevant to understanding and prioritizing water quality research related to geogenic constituents (Fig. 2). Broadly speaking, water availability is affected by both natural conditions and human alterations to freshwater resources, which includes both surface water and groundwater (Abbott et al., 2019; Evenson et al., 2013). Streams and groundwater can exhibit alteration or stress, such as water-level changes or water quality problems. Certain populations can be disproportionately affected by drinking water availability because of proximal contamination sources, lack of proximal water quality data, or historically overlooked factors such as having a private domestic well as the sole drinking water source, or other societal factors.

We used non-parametric correlation analysis and professional judgement to inform the selection of the 12 study variables described in Table 3, which represent relevant factors. The study variables include conventional physical and geochemical factors that relate to the distribution and mobilization of geogenic constituents, such as the distribution of elevated arsenic in groundwater or irrigation water use (Fig. 2). We also chose as study variables often overlooked variables related to societal factors associated with the effect of geogenic constituents on groundwater-sourced drinking water supplies. As described in more detail in the next sections, the study geospatial variables fall within the broad categories of *system condition or stress*, *human alteration*, and *historical disparity* (Fig. 2, Table 3).

**Table 3:** Variables used in ranking calculations. Additional detail provided in the data release (Qi et al., 2023)

Variable name and data source description (original data units; source reference)	Variable value assigned to candidate basins (variable units)	How higher percentile scores were assigned
*System condition or stress variables*		
1. As > 5: Probability of arsenic concentration exceeding 5 µg/L in groundwater used for domestic supply (probability; Lombard et al., 2021a)	Percent of area with a probability of As > 5 µg/L greater than 0.15 (percent of basin area)	Higher percentage of the area likely to have elevated arsenic
2. Shallow_brackish: Three-dimensional probability of brackish groundwater occurrence (predicted depth; Stanton et al., 2017)	Percent of area with predicted depth to brackish water 500 ft (152 m) or shallower (percent of basin area)	Higher percentage of the area likely to have shallow brackish groundwater
3. Mag_GW_change: Groundwater storage change measured by the Gravity Recovery and Climate Experiment (GRACE) (centimeters per year; Velpuri et al., 2019)	Absolute value of the mean rate of change (average magnitude in the basin, centimeters per year)	Larger change in groundwater storage (positive or negative change)
4. Mag_ppt_change: Change in modeled precipitation from 1971–2000 versus 2070–2099. Ensemble mean of 20 global climate models (millimeters per year; https://climate.northwestknowledge.net/MACA/)	Absolute value of mean change in precipitation (average magnitude in the basin, millimeters per year)	Larger change in precipitation (positive or negative change)
*Human alteration variables*		
5. %_GW_DW: Proportion of population served by publicly supplied groundwater, publicly supplied surface water, population served by each, and population served by private domestic wells (percent of GW public supply; Johnson et al., 2019, 2021, 2022)	Sum of the population served by groundwater divided by total population served (percent of basin population)	Higher percentage of the population served by groundwater as a drinking water source
6. Irr_WU: Total irrigation (groundwater and surface water) water use withdrawals (million gallons per day per county; Dieter et al., 2018)	County-level water use data apportioned by area to the candidate watershed (million gallons per day per basin)	Higher volume of irrigation water use
7. EPA_site_den: Number of sites regulated by USEPA (count; https://www.epa.gov/frs)	Density of EPA-regulated sites (average sites per square kilometer per basin)	Larger density of EPA-regulated sites
8. Mines: Number of non-aggregate mines (count; https://mrdata.usgs.gov/mrds/)	Sum of the number of non-aggregate mines (count of sites in the basin)	Larger number of non-aggregate mines
*Historical disparity variables*		
9. Dom_well_pop: Population served by domestic well water (people per square kilometer; Johnson et al., 2019)	Sum of the domestic well population (number of people served by domestic wells per basin)	Larger population served by domestic wells
10. Soc_dem: Sociodemographic measure calculated as [(% minority + % low-income)/2]. (median national percentile per block group; EJ Mapper at https://ejscreen.epa.gov/mapper/)	Census-block sociodemographic measure apportioned by area to the candidate watershed (median sociodemographic measure per basin)	Higher percentage of population minority or low income
11. Native_pop_den: Density of Native American population (population per block group; ESRI, 2021, USA Demographics and Boundaries 2021)	Census-block Native American population apportioned by area to the candidate watershed (average number of Native Americans per square kilometer per basin)	Higher density of Native American population
12. Data_gaps: Number of wells with a groundwater sample analyzed for trace elements (count; U.S. Geological Survey, 2019)	Sum of the number of wells with a groundwater sample analyzed for trace elements (− 1 * count of sampled wells per basin)	Larger data gap, as quantified by fewer wells with a trace element sample

### Variable value assignments to candidate basins

Each of the 12 variables was represented as point, raster, or polygon geospatial data (Qi et al., 2023). The spatial variables were apportioned to each candidate basin using various spatial summary techniques within a geographic information system (GIS) depending on the type of source data. For continuous raster-type data, zonal statistics tools were used to calculate means, medians, or other summary statistics. For categorical data such as land cover or county-based data, summary tools based on area-weighting were used to calculate the required statistic. For point data, summary statistics were calculated based on the points contained within each candidate basin boundary (Table S3). Variables were scaled (e.g., averaged, summed) across the candidate basins as appropriate for the variable (Table 3, Table S3). The companion data release provides additional detail about data sources and data processing methods (Qi et al., 2023).

### Candidate basin ranking process

For each of the 12 variables, the scaled variable values were rank-ordered across all 163 candidate drainage basins by using the *percentrank* function in Excel® by which they were ordered numerically from 1 to 163, then the rank-order divided by 163 to compute the percentile rank of the variable scaled from 0 (lowest percentile rank) to 1 (highest percentile rank), referred to as “variable percentile ranks” (Table S4). The percentile ranking step was taken to adjust for different units among variables and to avoid undue influence from outliers. The method also regularized the data, which may have masked some natural breaks or over-emphasized marginally different values in some of the data sets.

**Fig. 3:**
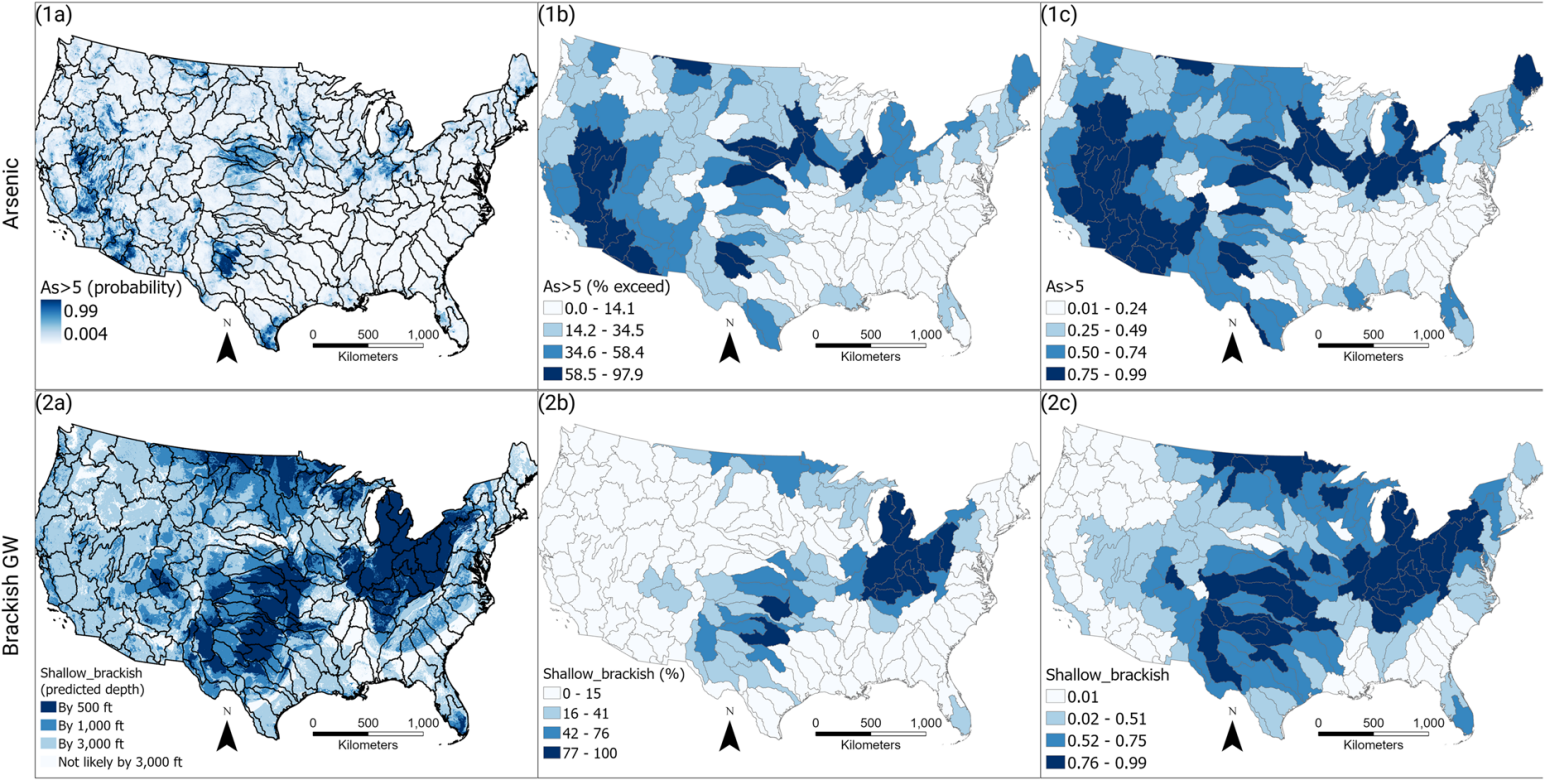
Illustration of the study basin ranking process for two representative variables: row 1 is a prediction of elevated arsenic in groundwater, and row 2 is likely the presence of brackish groundwater at a depth shallower than 500 ft (152 m). For each variable row, panel (a) shows the original national data set used for the variable; panel (b) shows the variable value assigned to each candidate basin; panel (c) shows the national percentile rank of each basin according to the single variable, with darker colors depicting higher percentile rank. Graphical presentations for all 12 variables are presented in supplemental Figure S2. All variables are described in Table 3 and in a data release (Qi et al., 2023)

Figure 3 illustrates graphically the candidate basin variable percentile ranking process for individual variables. Steps include apportioning the original data (Fig. 3a, Figure S2) to computed variable values for each candidate basin (Fig. 3b, Figure S2) and then to a national variable percentile rank for each candidate basin (Figs. 3c, S2). Results for two variables, elevated arsenic in groundwater and shallow brackish groundwater (described in Table 3 and in Qi et al. (2023)), are illustrated in rows 1 and 2 in Fig. 3. Comparable results for all 12 variables are illustrated in Figure S2.

**Fig. 4:**
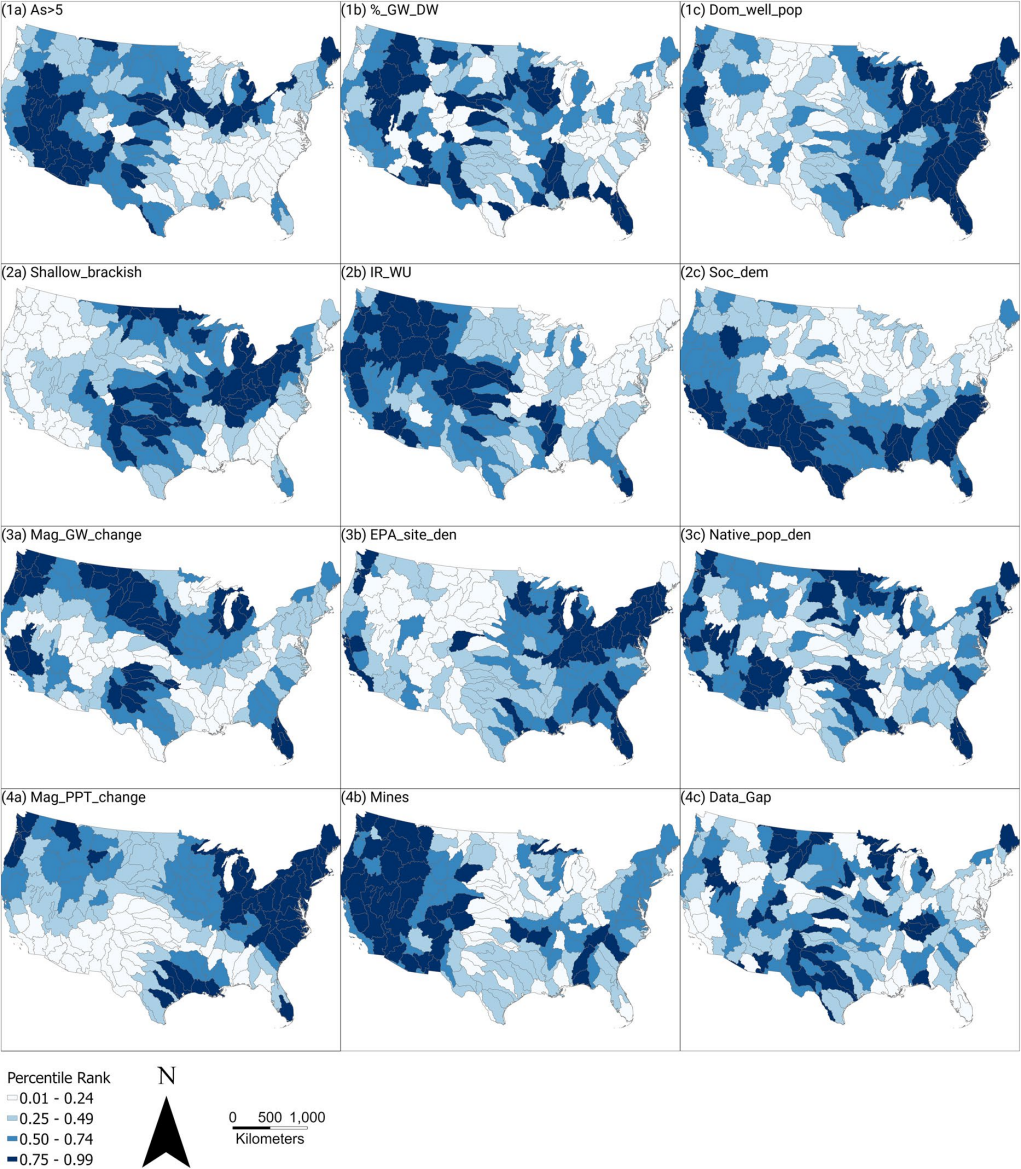
Maps of national percentile ranks of all variables for all candidate basins with darker colors depicting higher percentile rank. (a) panels, system condition or stress variables; (b) panels, human alteration variables; (c) panels, historical disparity variables. Variable descriptions are presented in Table 3

After each of the candidate basins was assigned each variable percentile rank (Fig. 4, Table S4), the 12 variable percentile ranks were summed for each basin to obtain a score for each basin. Each of the 12 variables carried equal weight in the basin scoring calculations. The basin scores were then ranked nationally from 1 to 163, with the highest score ranked as 1, referred to as basin “national ranks” (Table S4). Finally, basin “regional ranks” were determined by comparing basin scores within each of the 18 regions. The regional perspective provides a set of representative prioritized study basins that are relatively evenly distributed throughout the CONUS to support national water availability assessments (Van Metre et al., 2020). Regional basin ranks ranged from 1 to the number of candidate basins in a region (for example, Region 1 has 11 basins so basin regional ranks there range from 1 to 11; Region 3 has basin regional ranks from 1 to 3, and so on) (Table S2, Table S4).

### Study variables

Study variables are summarized in Table 3, each with the short variable name used for convenience, a variable description, the data source for the variable, and a description of criteria for higher percentile ranks for the variable. Additional descriptions and data source details are provided in supplementary Tables S3 and in Qi et al. (2023). The study variables are grouped into three categories. Two categories of variables, *system condition or stress* and *human alteration*, include relatively conventional physical and chemical factors that may be related to the distribution of geogenic constituents, such as the distribution of elevated arsenic in groundwater or irrigation water use (Fig. 2). We include a novel third category, historical disparity, to quantitatively include variables related to EJ considerations in our ranking method.

#### System condition or stress variables

Measured or modeled geochemical conditions and system stress responses to human alteration take into account patterns of constituent occurrence, existing water quality limitations, and areas of potential for exacerbation of contamination from geogenic constituents in drinking water aquifers (Ayotte et al., 2021b; Bondu et al., 2016; Lombard et al., 2021a; McMahon et al., 2016; Nordstrom, 2009; Stanton et al., 2017). Four variables were chosen to represent system condition or stress in the context of basin prioritization focused on geogenic constituents.

1. Probability of groundwater geogenic As concentrations higher than 5 μg/L (As > 5)—This is an indicator of potential geogenic limitations on water availability. The distributions of geogenic constituents incorporate a wide range of properties and occurrences throughout the CONUS (Tables 1 and 2, Table S1, Figure S1). Arsenic is only one of the many geogenic constituents with widely differing distributions (Table 1). Nonetheless, it is an important contaminant, and it was selected in part because its distribution is relatively well-characterized at the CONUS scale by measurements and modeling (Ayotte et al., 2017; Lombard et al., 2021a). The arsenic rankings are based on modeled concentration exceeding 5 µg/L, which is one-half the current MCL, thus emphasizing the importance of contaminant-source attribution efforts even where concentrations are below MCLs. Geogenic constituents are susceptible to uncertainties and potential changes in MCLs (U.S. Environmental Protection Agency, 2023) and existence of lower drinking water standards in some states (New Hampshire Department of Environmental Services, 2021; State of New Jersey, 2020). Current MCLs include cost–benefit and feasibility considerations for public water systems and do not consider the effects of contaminant mixtures. Elevated arsenic concentrations occur in numerous locations, related in part to underlying rock types, weathering intensity, and pumping history. The As model results are therefore useful as a proxy for conditions conducive to the mobilization of many geogenic constituents (Table 2, Table S1). For example, Scanlon et al. (2022) note that As violations (> MCL) in public water systems are similar in spatial distribution of violations related to radionuclides.
2. Probability of brackish groundwater expected within 500 ft of land surface (Shallow_brackish)—This is an indicator of potential limitations for depth of water well drilling for potable water. Brackish groundwater occurs at depths that are within the range of some drinking water wells in areas of the continental interior such as the Southern High Plains, Midwest, and Great Lakes regions; it also encompasses seawater intrusion into coastal aquifers in regions such as Florida and the Atlantic Coast. Brackish groundwater is commonly a source of geogenic constituents (e.g., Cl (chloride), SO_4_ (sulfate), NH_4_ (ammonium), CH_4_ (methane), Ra, Li (lithium), Sr (strontium), among others) that can affect drinking water availability by limiting the freshwater supply, especially where upwelling occurs because of intensive pumping. In addition, increasing salinization from upwelling saline groundwater, road salt application, or other surface sources, or from coastal seawater intrusion could all contribute to mobilizing geogenic constituents in drinking water aquifers or pipe corrosion releasing metals, depending on physical and geochemical conditions (McMahon et al., 2016; Stanton et al., 2017).
3. Magnitude of recent groundwater storage change (Mag_GW_change)—This is an indicator of measured groundwater level changes caused by climate variation and water use in the recent past (2003–2016) (Velpuri et al., 2019), which may affect geogenic constituent distributions in drinking water aquifers. Either higher or lower groundwater levels can mobilize geogenic constituents, depending on physical and geochemical conditions (Bondu et al., 2016; Lombard et al., 2021b; Nordstrom, 2009; Velpuri et al., 2019).
4. Magnitude of projected precipitation change because of climate change (Mag_ppt_change)—This is an indicator of model-based future climate change effects that could potentially alter geogenic constituent distributions in drinking water aquifers during the late twenty-first century (2070–2099). Either more or less precipitation (wetter or dryer climate conditions) could mobilize geogenic constituents in drinking water aquifers, depending on local physical and geochemical conditions and potential associated human adaptations (Aizebeokhai et al., 2017; Amanambu et al., 2020; Bondu et al., 2016; Lombard et al., 2021b; Nordstrom, 2009).

#### Human alteration variables

Human interactions with hydrologic and geochemical systems can alter the patterns of occurrence, mobility, transport, and fate of geogenic constituents in drinking water aquifers and surface water resources (Amanambu et al., 2020; Ayotte et al., 2011b; Ayotte et al., 2015; Borden et al., 2017; Degnan et al., 2020; Erickson et al., 2019; Lombard et al., 2021a; Nordstrom, 2011b; Scanlon et al., 2022). Four variables were chosen to represent human alteration in the context of basin prioritization focused on geogenic constituents.

5. Fraction of population with groundwater-supplied drinking water (%_GW_DW)—This is an indicator of public water supply (PWS) and private domestic supply water use needs. Dependence on groundwater for drinking water supply is an indicator of populations at potential risk from geogenic contaminants in drinking water aquifers (Belitz et al., 2015, 2022; Johnson et al., 2019, 2021, 2022).
6. Irrigation water use (Irr_WU)—This is an indicator of agricultural land use, a measure of groundwater and surface water withdrawals, and other hydrologic system alterations that can influence geogenic constituent mobilization (Böhlke, 2002; Dieter et al., 2018; Dillon, 2005; Fakhreddine et al., 2021). Irrigation water use is largely associated with arid and semi-arid settings in the western USA, but it is also prominent in other regions such as the Gulf Coast, Florida, and Atlantic Coast, in part related to the intensification of cropping practices. In arid regions, irrigation can flush large quantities of naturally accumulated constituents in soils (e.g., NO_3_^−^, SO_4_, ClO_4_^−^, other oxyanions), along with anthropogenic agricultural constituents (e.g., NO_3_^−^, Cl, SO_4_), to the water table. Irrigation can also increase the recharge rate and flux of oxidants (oxygen, NO_3_^−^) and ions to groundwater, causing enhanced oxidation and leaching of geogenic constituents from soils and aquifer materials. Increased water flow through the shallow system can change the geochemistry of the vadose zone and shallow groundwater by changing redox conditions, introducing anthropogenic contaminants that can influence geogenic constituent mobilization, or redistributing geogenic constituents (Böhlke, 2002; Dieter et al., 2018; Dillon, 2005; Fakhreddine et al., 2021).
7. Density of sites regulated by EPA (EPA_site_den)—This is an indicator of developed land use, potential releases of waste or wastewater, potential releases of landfill leachate, and potential chemical or petroleum spills. Impermeable surfaces and releases of wastewater or spills can change the geochemistry of the vadose zone and groundwater by changing redox conditions, pH, introducing anthropogenic contaminants that can influence geogenic constituent mobilization, or redistributing geogenic constituents (Borden et al., 2017; Cozzarelli et al., 2016, 2017, 2021; Repert et al., 2006).
8. Number of non-aggregate mines (Mines)—Non-aggregate mines can be loci where geogenic constituents brought to the surface can be mobilized by anthropogenic activity. Thus, this variable is an indicator of sites that can potentially release wastewater, waste rock, sludge, anthropogenic contaminants, acid drainage, or inorganics such as arsenic, selenium, copper, and lead (Table 2, Figure S1). Such releases or spills can cause substantial direct contamination of water resources. Releases can also change the geochemistry of the vadose zone and groundwater by changing redox conditions or pH, by introducing anthropogenic contaminants that can influence geogenic constituent mobilization, or by redistributing geogenic constituents (Nordstrom, 2009, 2011a, 2011b; Schmidt et al., 2012).

#### Historical disparity variables

There is growing recognition that certain populations historically have been excluded or under-represented in water resource research. Underserved populations, therefore, have experienced a disproportionate hazard from poor water quality (Allaire & Acquah, 2022; Munene & Hall, 2019; Ravalli et al., 2022; Scanlon et al., 2022; Schaider et al., 2019). Four variables were chosen to represent historical disparity in the context of basin prioritization focused on geogenic constituents.

9. Number of private domestic well users, with the estimate based in part on census information (Dom_well_pop)—This is an indicator of populations at risk from undocumented or unregulated geogenic contamination. Private domestic wells are often the sole source of drinking water (Johnson et al., 2019). Although USGS studies estimate that about 20% of private domestic wells contain at least one contaminant above a threshold (Ayotte et al., 2011a; DeSimone, 2009), private domestic well users may not perceive an existing water quality problem that is not observable or for other reasons (Munene & Hall, 2019; Schuitema et al., 2020). Unregulated private domestic well water quality is often of similar raw-water quality as public water systems (Spaur et al., 2021). Public water systems, however, are required to test water quality and provide treatment as necessary to ensure that water distributed meets water quality standards set by the EPA (Flanagan et al., 2015; Johnson et al., 2019; Malecki et al., 2017; Spaur et al., 2021; U.S. Environmental Protection Agency, 2018). There are no such requirements for self-supplied domestic well water quality.
10. Sociodemographic measure related to populations with low income and population of color (Soc_dem)—This is an indicator of potential current and historical disparity in drinking water quality (Nigra & Navas-Acien, 2020; Nigra et al., 2020, 2022; Ravalli et al., 2022; Schaider et al., 2019; Tanana et al., 2021). Studies show that lower-income, less educated people are also less likely to test or treat private domestic well water (Flanagan et al., 2015, 2016; Malecki et al., 2017).
11. Density of Native American population (Native_pop_den)—This is an indicator of where Native Americans comprise a substantial part of the population. The census historically has undercounted certain parts of the Native American population (Norris et al., 2012), and well water quality on Native American lands may be poorly characterized (Fillmore & Singletary, 2021). Although the Native American population is also represented in the Soc_dem variable, we include this separate variable representing the Native American population to help balance the historical underrepresentation of this demographic group in summaries based on the census, including the estimate of private domestic well users (Norris et al., 2012; Sobel et al., 2021; Tanana et al., 2021).
12. Data gaps as determined from relative numbers of groundwater samples analyzed by USGS for trace element concentrations (Data_gaps)—This is an indicator of how much quantitative trace element groundwater quality information was collected from 1988 to 2019 (Table 1 and supplemental information) (U.S. Geological Survey, 2019). Including a measure of what is known about water quality helps to account for historical disparity in knowledge of water quality hazards. A current DOI EJ priority is ensuring inclusive and equitable access and benefit from data, information, and science (U.S. Department of the Interior, 2022b) and community engagement (U.S. Department of the Interior, 2022a). For example, Southwestern US Native American communities have identified water quality information as their greatest need (Fillmore & Singletary, 2021).

## Results and discussion

### Overview

**Fig. 5:**
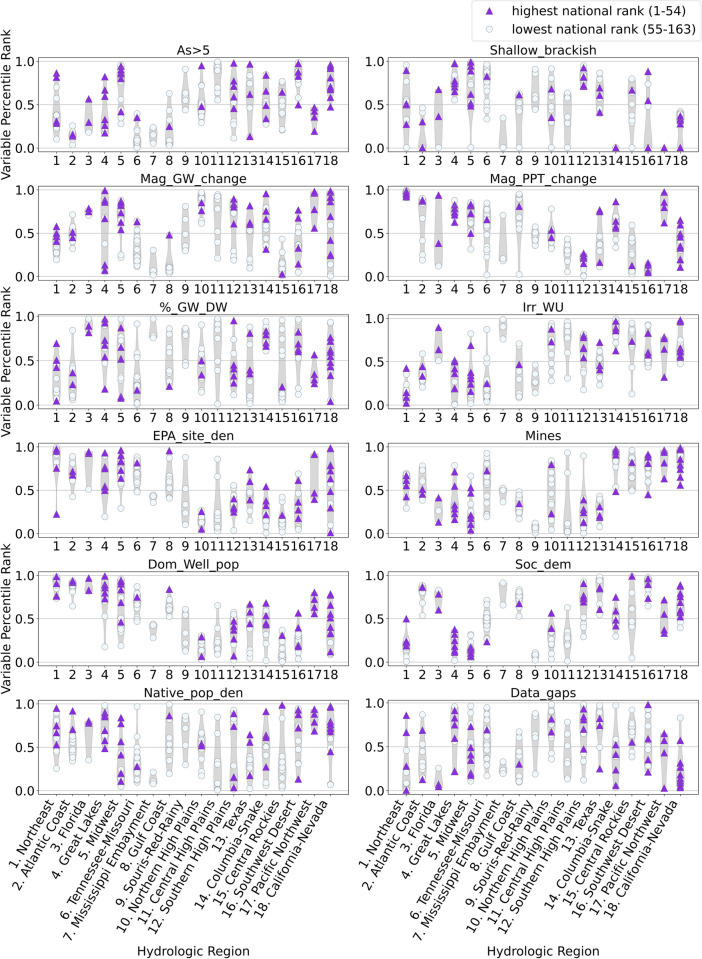
Percentile ranks of variable data, by region. Wider gray shading illustrates a higher proportion of the region’s data. Purple triangles indicate candidate basins with the highest national rank (Fig. 6a and Figure S3)

National maps of single-variable percentile ranks (Fig. 4) illustrate spatial patterns of the relative importance of individual variables across the CONUS. Plots of percentile ranks by region (Fig. 5) illustrate how different variables are distributed within and between the hydrologic regions. For example, for the As > 5 variable, Region 2 basin ranks are all in the bottom quartile; in contrast, Region 18 basin ranks are concentrated in the upper half. For some variables (e.g., Data_gaps), many regions have wide ranges of percentile ranks across candidate basins. In contrast, for other variables (e.g., DomWell_pop), many regions have smaller ranges of percentile ranks across candidate basins. Both Fig. 4 and Fig. 5 illustrate how individual variable percentile ranks can affect overall candidate basin scores differently.

**Fig. 6:**
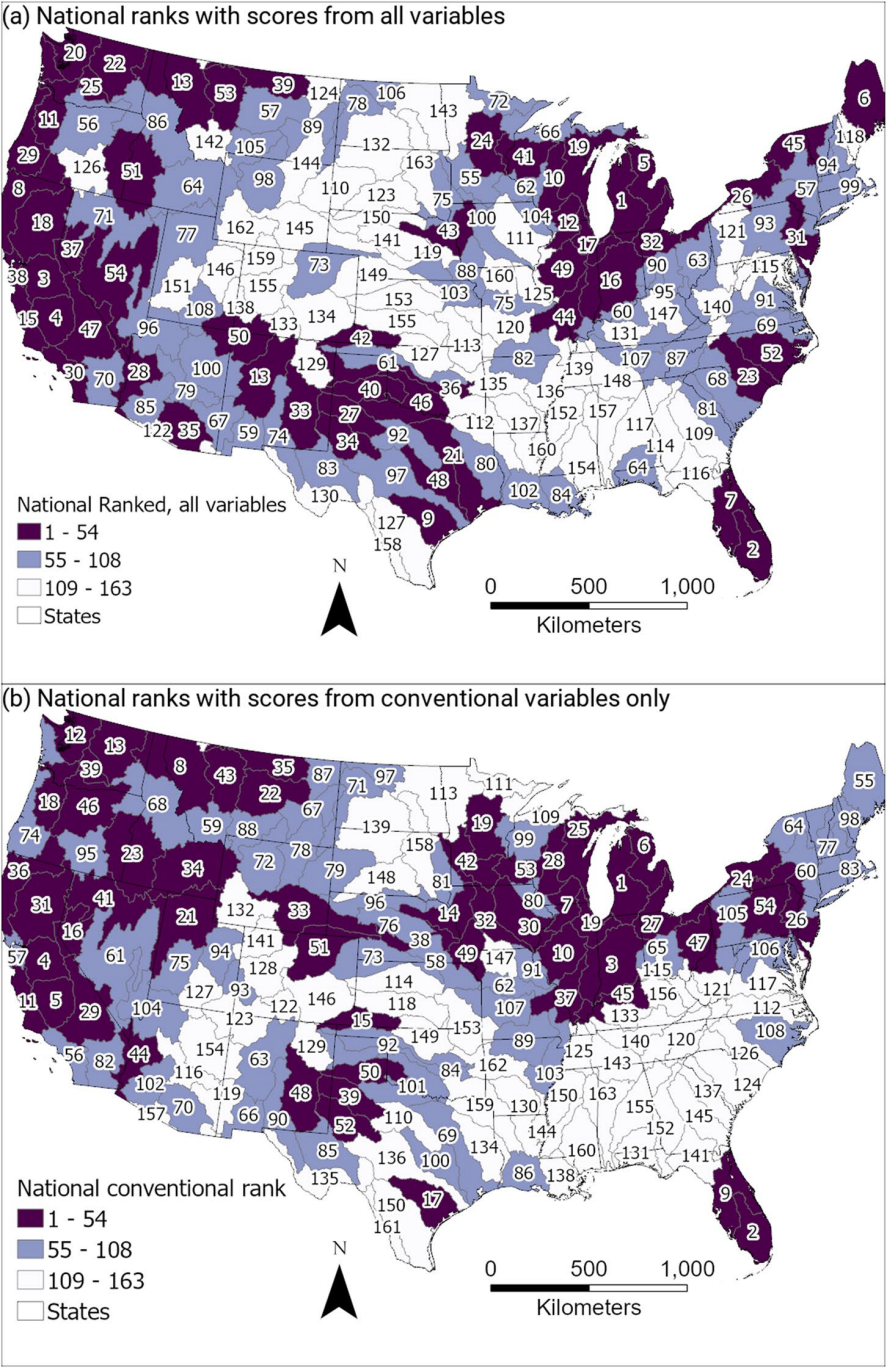
Maps of national candidate basin ranking with and without the historic disparity variable category, with darker colors indicating higher rank (higher priority). **a** National ranking that includes the novel historical disparity category of variables. **b** National ranking using only the conventional human alteration and system condition or stress categories of variables. Some basins in the northeast, eastern coast, and central southwest have elevated priority (1 being the highest rank and highest priority) with the inclusion of historic disparity variables. Detailed basin variable percentile ranks are provided in Figs. 4 and 5 and Table S4

Multi-variable national basin ranks are summarized in Fig. 6, which includes a comparison of results based on summing percentile ranks for variables in all three variable categories in contrast to results based on summing percentile ranks for only the two conventional variable categories (system response or stress, and human alteration). National multi-variable basin ranking in the context of selected principal aquifers (PA) is illustrated in Figure S3. Table S4 presents tabulated ranking details, including percentile ranks for each basin for each variable, overall summed basin scores, basin national ranks, and basin regional ranks. Regional rankings are presented in Figures S4 and S5 and Table S4.

### National perspective

A map of the national ranking of candidate basins across the CONUS indicates that the highest-ranking areas include clusters of basins in proximity to one another in the Northeast, Southeast, Midwest (especially near the Great Lakes), West, and the central part of the Southwest (Fig. 6A). Elevated concentrations of the most common geogenic contaminants vary across the hydrologic regions (Table 1). Most of the highest ranked candidate basins intersect with principal aquifers (Figure S3); about half intersect with or are wholly within the footprint of the glacial aquifer system. Some of the sandstone and carbonate aquifers in the Midwest also coincide with high-ranked basins. Much of the igneous and metamorphic aquifers in the Northeast and Northwest are also overlain by high-ranking basins. The unconsolidated sand and gravel aquifers in the West, central part of the Southwest, and Southeast also have substantial portions overlain by high-ranking basins.

Different variables have additive influence resulting in high multi-variable basin ranks in different parts of the country (Figs. 4 and 5, and S2). For example, in the eastern part of the country, the density of EPA sites and population using private domestic wells contribute to high basin ranks, whereas in the western part of the country, the number of mines and the sociodemographic measure variable tend to influence high ranking.

When the historical disparity category of variables is omitted from the analysis, the basin ranking method yields substantially different rankings in some parts of the country than when this novel variable category is included (Fig. 6A compared to 6B). The differences in rank of basins in three areas (northeasternmost portion of the Northeast region, central portion of the Atlantic Coast region, and portions of the Southern High Plains and Texas regions) stand out and are discussed in more detail in the next sections.

#### Northeastern Northeast region

Northeastern states are traditionally water-rich in both surface and groundwater (Fig. 1). Plentiful water has created some complacency with respect to the groundwater-sourced water supply that is increasingly being challenged by the impacts of precipitation events of high intensity followed by long periods of no precipitation (Flanagan et al., 2015). In the northeasternmost part of the Northeast Region, about 50% of the population uses private domestic wells for water supply (Johnson et al., 2019; Lombard et al., 2021a), which is a risk factor for exposure to geogenic constituents. Geogenic contaminants such as As and U are prevalent in private domestic wells in the area (Belitz et al., 2022; Teeple et al., 2021; U.S. Geological Survey, 2019) and generally increase in concentrations with increasing well depth (Flanagan et al., 2018).

In the Northeast region, the prevalence of As in groundwater, the density of EPA-ranked contamination sites, and the relatively high population using private domestic wells tend to influence the high-ranking basins in the region (Figs. 4 and 5). Increasingly, states are receiving reports of wells going dry or having insufficient water (Bellavance, 2022; Bidgood, 2016). Lower water levels also may impact the concentration of constituents such as As (Degnan et al., 2020; Lombard et al., 2021b). It is not clear how changing climatic conditions (temperature and precipitation) might affect changes in geogenic constituent mobilization or concentrations in the region (e.g., As).

In studies in and around the Northeast region, factors related to socioeconomic status, such as income, education, and cost of testing and treating well water, have been reported to inhibit the ability of private domestic well owners to take appropriate actions to ensure that their private domestic drinking water supplies are safe for consumption (Zheng & Ayotte, 2015). Education level, optimism bias (perception that your well water is better than your neighbors), inconvenience, and cost are all factors inhibiting domestic well owners (Zheng & Ayotte, 2015).

A study in Maine found that socioeconomic status was a factor. More educated and higher income households were more likely to have tested their well water, education was significantly associated with having tested in the last 5 years for As, and income was significantly associated with whether As was included in the most recent test (Flanagan et al., 2015). In Nova Scotia, Canada (immediately to the northeast of Maine), cost was a less significant factor than convenience, awareness, and perception; nevertheless, the adjusted odds of households taking action to improve well water safety was 2.5 (95% CI 1.2–5.4) times greater among those with a family income of $100,000 or more compared with those having a family income less than $25,000 (Chappells et al., 2015).

The EPA serves ten federally recognized tribes in the Northeast region states of Maine, Connecticut, Rhode Island, and Massachusetts. Many of the tribes obtain water from public water supplies, but some use private domestic wells. How water resources on Tribal lands are affected by disparity in the prioritization of water quality studies is not well known and there are opportunities for improvement in both our understanding of water quality on these lands and in communicating water quality issues to the communities affected (Figs. 4 and 6). For example, the Passamaquoddy Tribe has been plagued with poor-quality water that is sourced from surface water with high levels of organic constituents (Rogers, 2022). A proposed solution involves using groundwater for water supply, which has not yet been permitted (Feinberg, 2021; Rogers, 2022). Although this solution may address contaminants such as trihalomethane compounds found in the current supply, it could bring other concerns if the groundwater source has geogenic constituents, such as As, which are common in wells in this area (Rogers, 2022). The Passamaquoddy Tribe and other examples point to the need to address societal biases in studies related to drinking water supply.

#### Central Atlantic Coast region

Many candidate basins in southeastern states within the central Atlantic Coast region returned relatively low (national rank > 100) rankings for geogenic constituent prioritization, based solely on the more traditional water quality ranking variables (Fig. 6b). With the addition of the four historical disparity variables, however, basins in this region commonly ranked substantially higher (national rank 23–116) (Fig. 6a). This shift is partly because of the large number of private domestic drinking water wells highlighted in Figs. 4 and 5 with a 0.75–0.99 percentile rank. These wells are exempt from the Safe Drinking Water Act in the USA and less likely to be monitored. Geogenic constituents can be especially prone to co-occurrence because of shared geological sources, for example, As and fluoride in felsic rock aquifers (Rango et al., 2010) or V (vanadium) and Cr in mafic or ultra-mafic aquifers (Manning et al., 2015; Wright & Belitz, 2010). In North Carolina, the co-occurrence of As, U, V, and Cr has been widely documented in well water across the state (Coyte & Vengosh, 2020). Exposure to multiple constituents without clear guidance or coordinated monitoring systems may put communities at higher risk for health hazards, especially where personal monitoring may be less likely because of societal factors (Flanagan et al., 2015).

Superfund sites (one of the site types included in the variable EPA_site_den) are locations identified by the EPA that are candidates for remediation because of an immediate and significant public health and/or environmental risk. In addition to hazards from private domestic wells, communities of color and/or lower income in the southeastern USA are also at risk for proximity to Superfund sites, due in part to the higher density of these sites in this region (Figs. 4 and 5). For example, a South Carolina spatial analysis indicated that about 56% of African American people in South Carolina live near Superfund sites (Burwell-Naney et al., 2013). Furthermore, across all populations in South Carolina living below poverty, about 57% are in proximity to Superfund sites (Burwell-Naney et al., 2013). Our analysis also demonstrates a high concentration of EPA sites overlaps with a high density of native populations in some areas of the southeastern USA (Fig. 4). Proximity to Superfund sites has been linked to elevated cancer risk (Amin et al., 2018), further stressing disadvantaged communities.

Another source of geogenic contaminants in the region is coal production, including mining, which is elevated in Appalachia and parts of the southeast with a 0.5–0.99 percentile rank (Figs. 4 and 5). Coal contains many geogenic constituents, which vary by coal seam and underlying geology. Once the coal is burned, the waste products become enriched in geogenic constituents (Altıkulaç et al., 2022). Coal combustion residuals, such as coal ash, are stored in surface impoundments and landfills, which contain As, B (boron), Mn (manganese), Se, Mo (molybdenum), U, and other geogenics (Harkness et al., 2016; Izquierdo & Querol, 2012). Impoundments can leak, or large spills can occur such as the Kingston Tennessee Valley Authority coal ash spill in 2008 and the Dan River at Duke Energy spill in 2014 (Harkness et al., 2016; Ruhl et al., 2010).

The Atlantic Coast region and other southeastern communities are also susceptible to climate hazards such as flooding and hurricanes, compounding the risks of water quality stressors and potential geogenic contamination in part because of the many coal ash impoundments in areas prone to flooding and severe storms (Vengosh et al., 2019). Since 2000, seven hurricanes category 1 or higher have made landfall in North Carolina with damage estimates ranging from $5.3 to 27.8 billion (Smith, 2020). The effects of these storms on groundwater wells are studied primarily in terms of salinity (Anderson Jr & Lauer, 2008; Carlson et al., 2008; William, 2002), and there is a gap in knowledge surrounding geogenic constituent behavior during and post hurricane. Furthermore, coal combustion residual storage areas in the region are susceptible to flooding post hurricane, and the release of hazardous materials has been linked to events such as Hurricane Florence (Vengosh et al., 2019). Atlantic Coast region basins highlighted in Fig. 6 have an overall increased vulnerability to water availability and geogenic constituent contamination because of the predisposition to climate hazards in conjunction with societal factors (Cutter et al., 2003; Drakes et al., 2021). These risks paired with sociodemographic factors that rank in the top 50% percentile of regions analyzed here (Fig. 5) make these basins strong candidates for prioritized research with regard to geogenic constituents and water quality.

#### Central southwest Southern High Plains and Texas Regions

The central southwest area has long seen declining groundwater levels in the High Plains aquifer (underlying the High Plains regions), as groundwater withdrawals have increased for irrigation and other purposes (Council for Agricultural Science and Technology (CAST) (2019); Dieter et al., 2018). The vulnerability and limitation of groundwater resources in the area are reflected in high percentile ranks for irrigation water use, groundwater storage change, and shallow brackish groundwater variables; in New Mexico basins, high percentile ranks result from the number of mines (Fig. 4). Additionally, historical disparities are reflected in high percentile ranks for the Native American population, sociodemographic measures, and data gap variables (Fig. 4).

Historical U mines in New Mexico have left contaminated mine waste and contaminated groundwater on Native American (for example, Navajo Nation) lands and other areas (U.S. Environmental Protection Agency, 2011). Groundwater level declines exacerbate water availability stresses from both legacy contamination and geogenic groundwater salinity (brackish groundwater) (Timmons, 2013). Many basins in the region have limited groundwater data for trace elements (Data_gaps, Fig. 3 and 4, S2). In areas where samples have been collected, high proportions of samples exceed thresholds for As, Sr, and Se (Table 1) (Ayotte et al., 2003; Moore et al., 2022). Ravalli et al. (2022) show the central southwestern USA as an area of high concentrations of As, Ba, Cr, Se, and U in community water systems. There is a strong association between public supply and private domestic well As concentrations (Spaur et al., 2021). Scanlon et al. (2022) illustrate that small community water systems are more likely to have violations of drinking water standards for As and radionuclides, and these systems face increased economic and other challenges applying for assistance in establishing effective treatment systems. There is a negative relationship between median household income and compliance with the As standard (Scanlon et al., 2022).

### Regional perspective

In addition to the national ranking perspective described previously, candidate basins also were ranked within each of the 18 hydrologic regions by using the full set of variables (Figures S4 and S5, Table S4). The regional perspective provides a set of representative prioritized study basins that are relatively evenly distributed throughout the CONUS to support national water availability assessments (Van Metre et al., 2020). Regional ranking currently is being used by USGS to select Integrated Water Science (IWS) basins representing different regions (Figure S5). This study’s regional ranking results could augment and broaden planned research activities in selected IWS study basins such that geogenic contamination research is considered. Regional ranking can also result in elevated importance of certain basins in some regions as compared to national ranking.

As described in Erickson et al. (2023) and references therein, currently selected IWS basins (as of 2023) have water availability considerations related to geogenic constituents. For example, the Willamette River Basin (Pacific Northwest region, 17) has several geogenic contaminants of concern, including high As in private domestic well water and high Hg in fish downstream of an abandoned mine. In this analysis, the Willamette River Basin is ranked highly nationally (11th) and within the region. Likewise in this analysis, the Delaware River Basin (Northeast region, 1) ranked highly nationally (31st) and within the region, and numerous geogenic contamination issues have been identified, including trace elements (Al (aluminum), As, Mn), the radionuclide Rn-222, and saltwater intrusion into public and private domestic supply wells. Past study of the Illinois River Basin (Midwest region, 5) identified contamination issues from As, Mn, radionuclides (including ^222^Rn, ^226^Ra, ^228^Ra, ^210^Pb, ^210^Po) (Szabo et al., 2020), and salinity in the major drinking water aquifers. Another concern in this basin is the redistribution of metals and industrial chemicals in Chicago area dredge sediment land applied in agricultural areas (Erickson et al., 2023). This basin was ranked highly nationally (17th) and within the region. The Trinity-San Jacinto basin ranked highly nationally (21st) and ranked first in its region. Our study results reinforce this basin’s established research priorities, which include addressing past environmental injustice as well as the effects of climate change such as sea level rise and drought (Erickson et al., 2023). Our study offers a different perspective on water quality research priorities in current and future USGS research activities in IWS basins, other USGS research efforts, or other research groups.

### Limitations

We recognize that all ranking approaches will reflect choices regarding variable selection and variable manipulation, which are influenced by the objectives of the study and people involved. Thus, our prioritization scheme for water quality study basins related to limitations on water availability from geogenic contamination is likely incomplete. Many additional considerations or data sets could have been incorporated in the ranking, and new data sets will become available. Nonetheless, the process and approach can be applied to other combinations of existing or new data sets.

Our ranking and prioritization were focused on geogenic constituents that are commonly found at high concentrations nationally (Belitz et al., 2022; DeSimone, 2009; U.S. Geological Survey, 2019). Constituents that occur at high concentrations locally may have muted signals in CONUS-scale representations. Many constituents could not be considered due to a lack of CONUS-scale data. The uneven distribution of available data was incorporated into our ranking scheme by including a Data_gaps variable that prioritized candidate study basins with relatively few groundwater quality samples in the national data sets.

Arsenic concentration was chosen as a representative geogenic constituent variable in the current study because of its importance, widespread occurrence, and the availability of a national As distribution model (Lombard et al., 2021a). Many other geogenic constituents are also important nationally, and each constituent likely has a different distribution pattern related to its sources and geochemical conditions for mobility. For example, many geogenic constituents are cationic as opposed to anionic or neutral like As, with different implications for geochemical behavior and mobility (Figure S1). Improved estimates of distributions of more geogenic constituents at the national scale would permit a more complete prioritization of targeted investigations. The inclusion of more or different geogenic constituent models would likely change ranking results.

Current data availability, reliability, and resolution are inconsistent at the national scale. The variables evaluated in this study were selected for national coverage, and most variables rely on spatial statistical models that are based on more limited data sets. Because this is a CONUS-scale ranking, only data sets with complete CONUS-scale data availability were incorporated. Important information, such as locations of lead-bearing water delivery system components and fish consumption advisories because of elevated Hg or other metals, was not considered because CONUS-scale information was not available. The inclusion of variables related to water delivery or fish consumption would likely change ranking results.

National rankings of individual variables (Figs. 4 and 5, S2) can either reinforce each other (similar map patterns) or offset each other (different map patterns). Because many variables have contrasting map patterns, overall national and regional candidate basin ranking scores have limited ranges (scores summed across the 12 variables average 6.0 (standard deviation 0.7; range 4.2 to 8.1)). In addition, the procedure used to rank candidate basins nationally for individual variables was designed to minimize the effects of outliers with extreme individual variable values. This approach could mute the relative importance of individual variables across the basins. For example, the county-scale values for irrigation water use ranged from 0 to 1850 million gallons per day, though relatively few counties have irrigation water use exceeding 450 million gallons per day (Figure S2). The assignment of irrigation water use values to candidate basins and then subsequent percentile ranking of basins smoothed the distribution. Choosing a different type of variable apportionment to candidate basins would likely change ranking results.

The modified HUC4-scale basin consolidation of data for ranking in this study was designed to provide representative subregions for intensive USGS monitoring and research in support of a national assessment of water availability (Van Metre et al., 2020). It is also an exploratory study to test a process, see how useful the process might be, and identify limitations. Water quality research in any priority area is likely to involve studies at various other scales, including larger areas for geospatial statistical analyses and smaller areas for intensive local process studies. This study provides a national-scale perspective on prioritizing locations for water quality research with respect to geogenic constituents. It does not, however, define any priority for a specific type of research or specify the priority of geography at a scale smaller than a candidate basin. Local studies of hydrogeologic and biogeochemical processes commonly benefit from additional criteria such as previous knowledge, feasibility, and representativeness. This study can help inform choices about where to prioritize research, but any specific research project design would need to consider other factors and criteria.

## Summary and implications

This study presents a multi-dimensional perspective on the selection of geographically representative research sites at a scale that might be suitable for national water availability studies. In a companion study, Erickson et al. (2023) summarized four key knowledge gap topics and associated research opportunities specific to understanding geogenic constituent occurrences and effects on water availability: (1) geogenic constituent sources and distribution processes, (2) geogenic constituent distribution and risk, (3) anthropogenic activity effects on geogenic constituent distribution, and (4) climate change effects on geogenic constituent distribution (Table S5). Those knowledge gaps and research opportunities overlap with variables used in the current geographic ranking process and could inform future water quality-focused water availability research activities in prioritized basins.

Geogenic constituents are ubiquitous at elevated concentrations in water resources across the CONUS, commonly occurring at levels that exceed regulatory or advisory drinking water limits (Belitz et al., 2022; DeSimone et al., 2015). Although quantitative consideration of mixtures of geogenic constituents was beyond the scope of this study, our analysis and discussion also highlight the co-occurrence of multiple geogenic constituents at elevated but sub-regulatory individual concentrations in crucial drinking water aquifers across the USA (Table 1 and Fig. 1). Drinking water standards that are enforceable for public water systems (e.g., EPA MCLs) are developed based on human health considerations balanced with economics and technology, and there can be subthreshold health effects (Agathokleous et al., 2022). Therefore, it is important to recognize research prioritization benefits that can come from consideration of constituent concentrations below current drinking water thresholds, such as a better understanding of geochemical processes and controls, anticipating uncertainties and potential future changes in regulatory dose–response functions (U.S. Environmental Protection Agency, 2023) including effects of mixtures, potential for future concentration increases, recognition that the effects of mixtures are not well known, acknowledgement that drinking water thresholds are not enforceable for private domestic wells, and clues to potentially higher concentrations in under-represented (data-poor) areas.

Economic and racial disparities in drinking water quality are widely documented in recent research publications. Small public water systems have economic and other challenges in establishing and maintaining effective treatment systems to meet enforceable drinking water thresholds. Drinking water standards are not enforceable for self-supplied domestic well water, leaving private domestic well users vulnerable to geogenic contaminant hazards. Groundwater sample locations are unevenly distributed, so there are areas with little knowledge of groundwater quality but substantial populations relying on private domestic wells for drinking water. Our analysis illustrates that conventional physical–chemical variables and novel historical disparity variables can be considered together using a quantitative method to geographically prioritize water quality research. The analysis also illustrates that prioritization can shift depending upon the variables considered, and different regions and considerations can be highlighted and revealed through a more inclusive selection of variables. Water supply issues affect communities in socially disadvantaged places—indigenous communities, poor urban communities, and other parts of rural America—and studies that focus on those communities and their issues could help. This study demonstrates a quantitative method for considering societal factors in research prioritization processes.

Geogenic constituents are only a subset of the many water quality constituents that affect groundwater and surface water resource availability and ecosystem health. This study provides a unique perspective on water quality research priorities in current and future research objectives with respect to geogenic constituents. Parallel water availability research prioritization schemes are in development for other constituents, such as temperature, suspended sediment, salinity, nutrients, and organic contaminants of emerging concern. The consideration of multiple prioritization schemes for multiple constituents and societal factors is likely to yield the most comprehensive results for current and future research. We focused on geogenic constituents because of their national-scale prevalence, and the consideration of societal factors relevant to evaluating EJ is likely to increase equity and reduce bias in any type of research that addresses water availability concerns. This scalable methodology could be globally applicable to any country or region in which relevant spatial data sets are available. The wider availability of relevant spatial data sets (geochemistry, climate, societal factors, etc.) would expand potential application.

### Supplementary Information

Below is the link to the electronic supplementary material.

Supplementary file1 (PDF 3976 KB)

Supplementary file2 (XLSX 114 KB)

## Data Availability

The data sets analyzed during the current study are available in the data release Qi et al. (2023), hosted by ScienceBase, https://www.sciencebase.gov/catalog/.
