# Supplementary material for: Prioritizing water availability study settings to address geogenic contaminants and related societal factors
Source: Environ Monit Assess. 2024 Feb 24;196(3):303. doi: 10.1007/s10661-024-12362-2 (PMC10894127; doi:10.1007/s10661-024-12362-2)
Supplement: Supplementary file 1 — Supplementary file1 (PDF 3976 KB) [file 10661_2024_12362_MOESM1_ESM.pdf]

Supplemental information for "Prioritizing water availability study settings to address geogenic contaminants and related societal factors"

Melinda L Erickson, Corresponding author. U.S. Geological Survey, 2280 Woodale Drive, Mounds View, MN 55112, United States. merickso@usgs.gov, ORCID 0000-0002-1117-2866

Craig J. Brown, U.S. Geological Survey, 101 Pitkin Street, East Hartford, CT 06108, United States. cjbrown@usgs.gov, 0000-0002-3858-3964

Elizabeth J. Tomaszewski, U.S. Geological Survey, 12201 Sunrise Valley Dr, Reston, VA 20192, United States. etomaszewski@usgs.gov, 0000-0003-3758-8322

Joseph D. Ayotte U.S. Geological Survey, 331 Commerce Way, Pembroke, NH 03275, United States. jayotte@usgs.gov, 0000-0002-1892-2738

J.K. Böhlke U.S. Geological Survey, 12201 Sunrise Valley Dr, Reston, VA 20192, United States. jkbohlke@usgs.gov, 0000-0001-5693-6455

Douglas B Kent U.S. Geological Survey, 345 Middlefield Rd, Menlo Park, CA 94025, United States. dbkent@usgs.gov, 0000-0003-3758-8322

Sharon Qi U.S. Geological Survey, 601 SW 2nd Ave. Suite 1950, Portland, OR 97204, United States. slqi@usgs.gov, 0000-0001-7278-4498

List of supplemental figures

Figure S1. Geogenic constituents, grouped by mobilization processes, sources, and conditions.

Figure S2. Illustration of the process of computing a value for each candidate basin from original data, along with the resulting percentile ranking of basins.

Figure S3. Map of national candidate basin ranking with selected principal aquifers for context of groundwater-source drinking water supply.

Figure S4. Percentile ranks of variable data, by region.

Figure S5. Map of regional candidate basin ranking using all variable categories.

List of supplemental tables

Table S1. Average abundance of selected geogenic elements in the upper crust, the equivalent pore-water concentration, human health benchmark, and the percent release to achieve exceedance.

Table S2. Candidate basins for the contiguous United States (from Van Metre et al. (2020), Table S1).

Table S3. Candidate basin calculated values for variables considered in ranking. Variable descriptions and their data sources are summarized in Table 3 with more detail in the data release (Qi et al., 2023).

Table S4. Percentile ranks, score (sum of variable percentile ranks), and final national and regional ranks by hydrologic region for candidate basins.

Supplemental text

We define geogenic water-quality constituents as any chemical or isotope that has a natural source (e.g., soil leaching, water-rock interaction, atmospheric deposition). Many geogenic constituents are, however, also manufactured or concentrated by humans. Although geogenic constituents commonly

occur in water because of natural processes, their distribution and concentration can be affected by human activities (e.g., mining, dispersal of mineral products, irrigation, pumping practices). Effects of human activities can be direct (e.g., application of fertilizers on the land surface) or indirect (e.g., alteration of geochemical or hydrologic conditions, which in turn affects constituent solubility or mobility).

Regional water quality summaries provided in Table 1 were compiled from samples in the U.S. Geological Survey National Water Information System data, 1988 – 2019 (U.S. Geological Survey, 2019). Site IDs are provided in the comma-delimited text file “Site_IDs” in the data release (Qi et al., 2023). For sites with more than one sample in the date range, the most recent sample was used. Sites were assigned to a region based on site location, so there are unequal numbers of samples across the regions (Table S3, sum by region the field “Count of wells with trace element samples”). Not all samples were analyzed for each constituent, so there are unequal numbers of samples for individual constituents. Concentrations in Table 1 and Table S1 were compared to relevant human health benchmarks (Norman et al., 2018; State of California, 2000; U.S. Environmental Protection Agency, 2018). Lithium is presented relative to two human health benchmarks, consistent with two other recent national-scale presentations of lithium in groundwater (Belitz et al., 2015; Lindsey et al., 2021).

Details about the selected variables are presented in Section 2.3 of the manuscript, in Table 3, and in Qi et al. (2023). A brief summary is provided here.

System condition or stress variables

- Probability of groundwater geogenic arsenic concentrations higher than 5 micrograms per liter (µg/L) [As>5]
- Probability of brackish groundwater expected within 500 feet of land surface [Shallow_brackish]
- Magnitude of recent groundwater storage change [Mag_GW_change]
- Magnitude of projected precipitation change due to climate change [Mag_ppt_change]

Human alteration variables

- Percent of population with groundwater supplied drinking water [%_GW_DW]
- Irrigation water use [Irr_WU]
- Density of sites regulated by USEPA [EPA_site_den]
- Number of non-aggregate mines [Mines]

Historic disparity variables

- Number of private domestic well users, with the estimate based in part on census information [Dom_well_pop]
- Sociodemographic measure considering populations with low income and population of color [Soc_dem]
- Density of Native American population [Native_pop_den]
- Data gaps as measured by the number of groundwater samples analyzed by USGS for trace element concentrations [Data_gaps]

Any use of trade, firm, or product names is for descriptive purposes only and does not imply endorsement by the U.S. Government.

Cited references

Appelo, C. A. J., Postma, D., & eds. (2005). *Geochemistry, Groundwater and Pollution, 2nd Edition*. CRC Press. https://doi.org/10.1201/9781439833544

Behera, S. N., Sharma, M., Aneja, V. P., & Balasubramanian, R. (2013). Ammonia in the atmosphere: a review on emission sources, atmospheric chemistry and deposition on terrestrial bodies. *Environmental Science and Pollution Research*, *20*(11), 8092-8131. https://doi.org/10.1007/s11356-013-2051-9

Belitz, K., Fram, M., Lindsey, B. D., Stackelberg, P. E., Bexfield, L. M., Kingsbury, J. A., Johnson, T. D., Jurgens, B. C., McMahon, P. B., & Dubrovsky, N. M. (2022). The quality of groundwater used for public supply in the continental United States: A comprehensive assessment. *Environmental Science&Technology - Water*. https://pubs.acs.org/doi/10.1021/acsestwater.2c00390

Belitz, K., Fram, M. S., & Johnson, T. D. (2015). Metrics for Assessing the Quality of Groundwater Used for Public Supply, CA, USA: Equivalent-Population and Area. *Environmental Science & Technology*, *49*(14), 8330-8338. https://doi.org/10.1021/acs.est.5b00265

Böhlke, J.-K. (2002). Groundwater recharge and agricultural contamination. *Hydrogeology Journal*, *10*(1), 153-179. https://doi.org/10.1007/s10040-001-0183-3

Borch, T., Kretzschmar, R., Skappler, A., Van Cappellen, P., Ginder-Vogel, M., Voegelin, A., & Campbell, K. (2010). Biogeochemical redox processes and their impact on contaminant dynamics. *Environmental Science and Technology*, *44*(1), 15-23. http://www.scopus.com/inward/record.url?eid=2-s2.0-75349085401&partnerID=40&md5=8a1aaf76f183bdf4fd7e4774eda40e9f

Champ, D. R., Gulens, J., & Jackson, R. E. (1979). Oxidation–reduction sequences in ground water flow systems. *Canadian Journal of Earth Sciences*, *16*(1), 12-23. https://doi.org/10.1139/e79-002

Chapelle, F. H., & Lovley, D. R. (1992). Competitive Exclusion of Sulfate Reduction by Fe(lll)-Reducing Bacteria: A Mechanism for Producing Discrete Zones of High-Iron Ground Water. *Groundwater*, *30*(1), 29-36. https://doi.org/https://doi.org/10.1111/j.1745-6584.1992.tb00808.x

Coyte, R. M., & Vengosh, A. (2020). Factors Controlling the Risks of Co-occurrence of the Redox-Sensitive Elements of Arsenic, Chromium, Vanadium, and Uranium in Groundwater from the Eastern United States. *Environmental Science & Technology*, *54*(7), 4367-4375. https://doi.org/10.1021/acs.est.9b06471

Cozzarelli, I. M., Kent, D. B., Briggs, M., Engle, M. A., Benthem, A., Skalak, K. J., Mumford, A. C., Jaeschke, J., Farag, A., Lane, J. W., & Akob, D. M. (2021). Geochemical and geophysical indicators of oil and gas wastewater can trace potential exposure pathways following releases to surface waters. *Science of The Total Environment*, *755*, 142909-142909. https://doi.org/https://doi.org/10.1016/j.scitotenv.2020.142909

Cravotta, C. A. (2008). Dissolved metals and associated constituents in abandoned coal-mine discharges, Pennsylvania, USA. Part 2: Geochemical controls on constituent concentrations. *Applied Geochemistry*, *23*(2), 203-226. https://doi.org/https://doi.org/10.1016/j.apgeochem.2007.10.003

Davis, J., & Kent, D. (1990). Surface Complexation Modeling in Aqueous Geochemistry. *Rev. Mineral.*, *23*.

Davis, J. A., Kent, D. B., Coston, J. A., Hess, K. M., & Joye, J. L. (2000). Multispecies reactive tracer test in an aquifer with spatially variable chemical conditions. *Water Resources Research*, *36*(1), 119-134. https://doi.org/https://doi.org/10.1029/1999WR900282

Dzombak, D. A., & Morel, F. M. M. (1990). *Surface Chemical Modeling: Hydrous Ferric Oxide*. John Wiley.

Erickson, M. L., Elliott, S. M., Brown, C. J., Stackelberg, P. E., Ransom, K. M., Reddy, J. E., & Cravotta, C. A. (2021). Machine-Learning Predictions of High Arsenic and High Manganese at Drinking Water

Depths of the Glacial Aquifer System, Northern Continental United States. *Environmental Science & Technology*, *55*(9), 5791-5805. https://doi.org/10.1021/acs.est.0c06740

Farag, A. M., Harper, D. D., Cozzarelli, I. M., Kent, D. B., Mumford, A. C., Akob, D. M., Schaeffer, T., & Iwanowicz, L. R. (2022). Using Biological Responses to Monitor Freshwater Post-Spill Conditions over 3 years in Blacktail Creek, North Dakota, USA. *Archives of Environmental Contamination and Toxicology*, *83*(3), 253-271. https://doi.org/10.1007/s00244-022-00943-6

Gerson, J. R., Szponar, N., Zambrano, A. A., Bergquist, B., Broadbent, E., Driscoll, C. T., Erkenswick, G., Evers, D. C., Fernandez, L. E., Hsu-Kim, H., Inga, G., Lansdale, K. N., Marchese, M. J., Martinez, A., Moore, C., Pan, W. K., Purizaca, R. P., Sánchez, V., Silman, M., . . . Bernhardt, E. S. (2022). Amazon forests capture high levels of atmospheric mercury pollution from artisanal gold mining. *Nature Communications*, *13*(1), 559. https://doi.org/10.1038/s41467-022-27997-3

Harkness, J. S., Dwyer, G. S., Warner, N. R., Parker, K. M., Mitch, W. A., & Vengosh, A. (2015). Iodide, Bromide, and Ammonium in Hydraulic Fracturing and Oil and Gas Wastewaters: Environmental Implications. *Environmental Science & Technology*, *49*(3), 1955-1963. https://doi.org/10.1021/es504654n

Hatzinger, P. B., Böhlke, J. K., Sturchio, N. C., Izbicki, J., & Teague, N. (2018). Four-dimensional isotopic approach to identify perchlorate sources in groundwater: Application to the Rialto-Colton and Chino subbasins, southern California (USA). *Applied Geochemistry*, *97*, 213-225. https://doi.org/https://doi.org/10.1016/j.apgeochem.2018.08.020

Hinkle, S. R., Böhlke, J. K., Duff, J. H., Morgan, D. S., & Weick, R. J. (2007). Aquifer-scale controls on the distribution of nitrate and ammonium in ground water near La Pine, Oregon, USA. *Journal of Hydrology*, *333*(2), 486-503. https://doi.org/https://doi.org/10.1016/j.jhydrol.2006.09.013

Jurgens, B. C., Parkhurst, D. L., & Belitz, K. (2019). Assessing the Lead Solubility Potential of Untreated Groundwater of the United States. *Environmental Science & Technology*, *53*(6), 3095-3103. https://doi.org/10.1021/acs.est.8b04475

Kolker, A., Engle, M. A., Peucker-Ehrenbrink, B., Geboy, N. J., Krabbenhoft, D. P., Bothner, M. H., & Tate, M. T. (2013). Atmospheric mercury and fine particulate matter in coastal New England: Implications for mercury and trace element sources in the northeastern United States. *Atmospheric Environment*, *79*, 760-768. https://doi.org/https://doi.org/10.1016/j.atmosenv.2013.07.031

Lindsey, B. D., Belitz, K., Cravotta, C. A., Toccalino, P. L., & Dubrovsky, N. M. (2021). Lithium in groundwater used for drinking-water supply in the United States. *Science of The Total Environment*, *767*, 144691. https://doi.org/https://doi.org/10.1016/j.scitotenv.2020.144691

Lovley, D. R. (1991). Dissimilatory Fe(III) and Mn(IV) reduction. *Microbiological Reviews*, *55*(2), 259-287. https://doi.org/doi:10.1128/mr.55.2.259-287.1991

Lytle, D. A., & Schock, M. R. (2005). Formation of Pb(IV) oxides in chlorinated water. *Journal AWWA*, *97*(11), 102-114. https://doi.org/https://doi.org/10.1002/j.1551-8833.2005.tb07523.x

McMahon, P. B., Böhlke, J. K., Dahm, K. G., Parkhurst, D. L., Anning, D. W., & Stanton, J. S. (2016). Chemical Considerations for an Updated National Assessment of Brackish Groundwater Resources. *Groundwater*, *54*(4), 464-475. https://doi.org/https://doi.org/10.1111/gwat.12367

McMahon, P. B., & Chapelle, F. H. (2008). Redox Processes and Water Quality of Selected Principal Aquifer Systems. *Groundwater*, *46*(2), 259-271. https://doi.org/https://doi.org/10.1111/j.1745-6584.2007.00385.x

Nordstrom, D. K., Blowes, D. W., & Ptacek, C. J. (2015). Hydrogeochemistry and microbiology of mine drainage: An update. *Applied Geochemistry*, *57*, 3-16. https://doi.org/https://doi.org/10.1016/j.apgeochem.2015.02.008

Norman, J. E., Toccalino, P. L., & Morman, S. A. (2018). *Health-Based Screening Levels for evaluating water-quality data (2d ed.). U.S. Geological Survey web page*. https://water.usgs.gov/water-resources/hbsl/

Qi, S. L., Baker, N. T., Johnson McKee, A., & Milinic, B. (2023). *Selected environmental characteristics for a national gap analysis of water-quality understanding and predictive capabilities to prioritize watersheds for research and monitoring needs: U.S. Geological Survey data release*. https://doi.org/10.5066/P9KFU815

Ram, R., Vaughan, J., Etschmann, B., & Brugger, J. (2019). The aqueous chemistry of polonium (Po) in environmental and anthropogenic processes. *Journal of Hazardous Materials*, *380*, 120725. https://doi.org/https://doi.org/10.1016/j.jhazmat.2019.06.002

Rudnick, R. L., & Gao, S. (2003). Composition of the continental crust. *Treatise on Geochemistry*, *3*, 1-64.

Seiler, R. L., Stillings, L. L., Cutler, N., Salonen, L., & Outola, I. (2011). Biogeochemical factors affecting the presence of 210Po in groundwater. *Applied Geochemistry*, *26*(4), 526-539. https://doi.org/https://doi.org/10.1016/j.apgeochem.2011.01.011

Selin, H., & Selin, N. E. (2022). From Stockholm to Minamata and beyond: Governing mercury pollution for a more sustainable future. *One Earth*, *5*(10), 1109-1125. https://doi.org/https://doi.org/10.1016/j.oneear.2022.09.001

Shi, Z., & Stone, A. T. (2009). PbO2(s, Plattnerite) Reductive Dissolution by Aqueous Manganous and Ferrous Ions. *Environmental Science & Technology*, *43*(10), 3596-3603. https://doi.org/10.1021/es8034686

Shi, Z., & Stone, A. T. (2009). PbO_2(s, plattnerite) reductive dissolution by natural organic matter: Reductant and inhibitory subfractions. *Environmental Science and Technology*, *43*(10), 3604-3611. https://doi.org/10.1021/es802441g

Soeder, D. J., & Miller, C. V. (2003). *Ground-water contamination from lead shot at Prime Hook National Wildlife Refuge, Sussex County, Delaware* [Report](2002-4282). (Water-Resources Investigations Report, Issue. https://doi.org/10.3133/wri024282

State of California. (2000). *Proposed Notification Level for Vanadium*. Sacramento, CA Retrieved from https://oehha.ca.gov/water/chemicals/vanadium

Stets, E. G., Lee, C. J., Lytle, D. A., & Schock, M. R. (2018). Increasing chloride in rivers of the conterminous U.S. and linkages to potential corrosivity and lead action level exceedances in drinking water. *Science of The Total Environment*, *613-614*, 1498-1509. https://doi.org/https://doi.org/10.1016/j.scitotenv.2017.07.119

Szabo, Z., Stackelberg, P., & Cravotta, C. A. (2020). Occurrence and geochemistry of lead-210 and polonium-210 radionuclides in public-drinking-water supplies from principal aquifers of the United States. *Environmental Science & Technology*, *54*(12), 7236-7249. https://doi.org/10.1021/acs.est.0c00192

U.S. Environmental Protection Agency. (2018). *2018 Edition of the Drinking Water Standards and Health Advisories Tables*. Retrieved from https://www.epa.gov/sites/default/files/2018-03/documents/dwtable2018.pdf

U.S. Geological Survey. (2000). *Ground Water Atlas of the United States* [Report](730). (Hydrologic Atlas, Issue. U. S. G. Survey. https://doi.org/10.3133/ha730

U.S. Geological Survey. (2019). *National Water Information System* [WWW Document]. http://waterdata.usgs.gov/nwis

Van Metre, P. C., Qi, S., Deacon, J., Dieter, C., Driscoll, J. M., Fienen, M., Kenney, T., Lambert, P., Lesmes, D., Mason, C. A., Mueller-Solger, A., Musgrove, M., Painter, J., Rosenberry, D., Sprague, L., Tesoriero, A. J., Windham-Myers, L., & Wolock, D. (2020). Prioritizing river basins for intensive monitoring and assessment by the US Geological Survey. *Environmental Monitoring and Assessment*, *192*(7), 458. https://doi.org/10.1007/s10661-020-08403-1

Vengosh, A., Coyte, R. M., Podgorski, J., & Johnson, T. M. (2022). A critical review on the occurrence and distribution of the uranium- and thorium-decay nuclides and their effect on the quality of groundwater. *Science of The Total Environment*, *808*, 151914. https://doi.org/https://doi.org/10.1016/j.scitotenv.2021.151914

Vroblesky, D. A., & Chapelle, F. H. (1994). Temporal and spatial changes of terminal electron-accepting processes in a petroleum hydrocarbon-contaminated aquifer and the significance for contaminant biodegradation. *Water Resources Research*, *30*(5), 1561-1570. https://doi.org/https://doi.org/10.1029/94WR00067

Supplemental Figures

Figure S1. Geogenic constituents, grouped by mobilization processes, sources, and conditions. Processes depicted in Panels (A) through (F) described below. Elements described in Table S1.

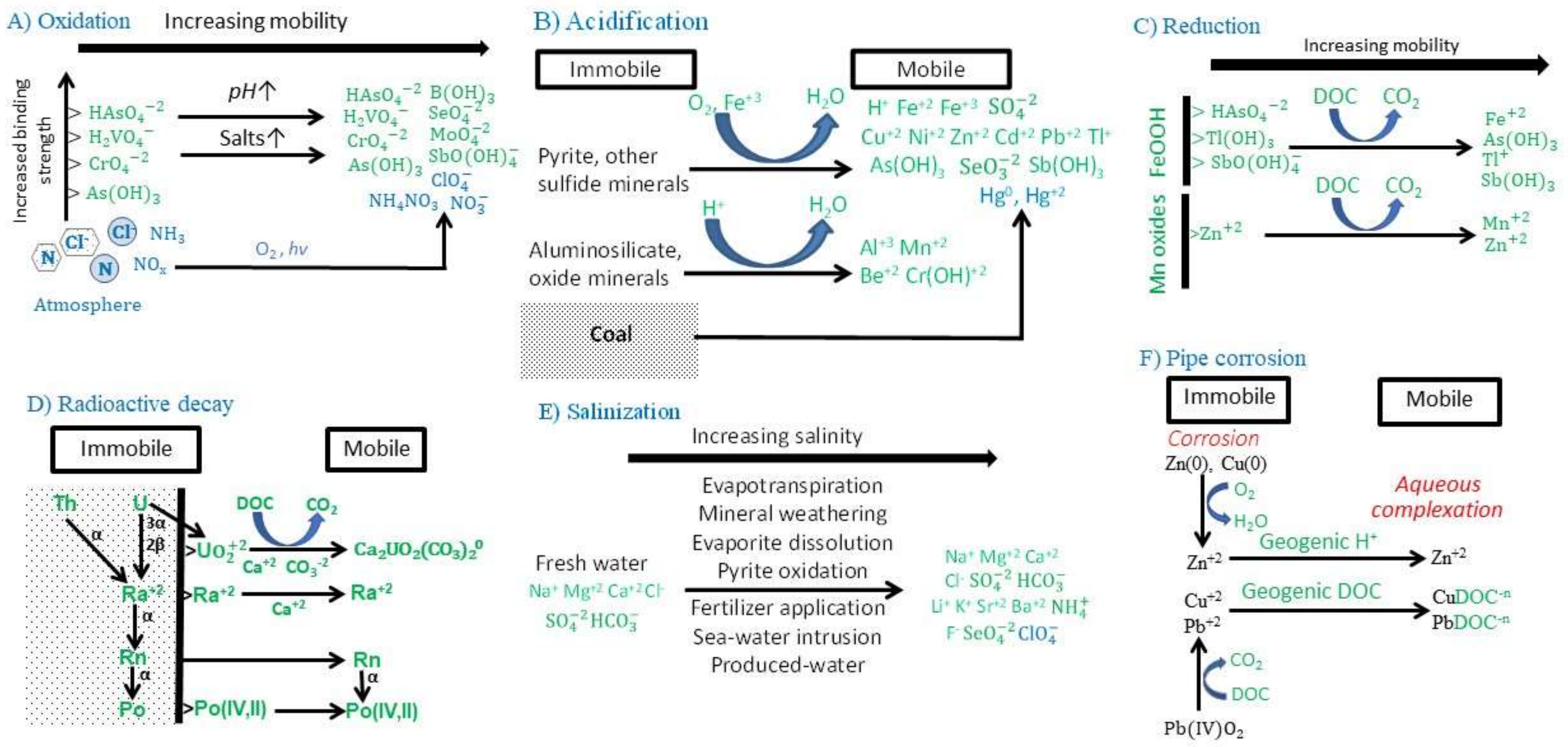

Color coding of elements/species. Green: aqueous and sediment-bound geogenic constituents. Blue: geogenic constituents influenced by atmospheric processes. Black: anthropogenic.

A) Oxidation. Mobility increases as the extent of sorption decreases. For many oxyanions, the extent of sorption decreases with increasing pH and dissolved salt concentrations (Appelo et al., 2005; Borch et al., 2010; Coyte & Vengosh, 2020; Davis & Kent, 1990; Davis et al., 2000; Dzombak & Morel, 1990; Rudnick & Gao, 2003). $H_2AsO_4^{-2}$, $B(OH)_3$, ClO_4^-, SeO_4^{-2}, MoO_4^{-2}, $SbO(OH)_4^-$, and Ca-UO_2^{2+}-CO_3 species are weakly sorbed and mobile across a wide range of conditions. NO_3^- and ClO_4^- are derived from mined nitrate fertilizers and from atmospheric processes that oxidize naturally occurring Cl^- and nitrogen species, primarily NO and NO_2 (NO_x), which combine with NH_3 on particulates (hexagons) and in aerosols (circles) (Behera et al., 2013). All other constituents shown are derived primarily from mineral weathering reactions. In this and all subsequent panels, sediment bound species (sorbed and in solids) are represented by a ">" symbol.

B) Acidification. Oxidation of Fe sulfide minerals such as pyrite yields sulfuric acid, resulting in acidic conditions where many metals (e.g., Cu, Pb), metalloids (e.g., As), and non-metals (e.g., Se) are mobile (Cravotta, 2008; Nordstrom et al., 2015). Pyrite can contain minor to trace amounts of As, Cd, Cu, Ni, Pb, Sb, and Tl. The acid produced (H^+) promotes dissolution of aluminosilicate and oxide minerals, resulting in the accumulation of constituents like Al, Be, Cr, and Mn, which are mobile at low pH (Nordstrom et al., 2015). Mercury has additional sources, including atmospheric deposition from sources such as coal-fired power plants, smelting, and gold mining (Gerson et al., 2022; Kolker et al., 2013; Selin & Selin, 2022).

C) Reduction. Inputs of labile dissolved organic carbon (DOC) or other electron donors can reduce Fe(III) oxyhydroxides and Mn oxides, mobilizing Fe(II), Mn(II), and constituents that preferentially sorb on these minerals (Borch et al., 2010; Erickson et al., 2021; Lovley, 1991). Redox zones result from the sequence of microbially catalyzed terminal electron accepting processes (oxidation of organic matter, the electron donor, coupled to the reduction of electron acceptors) along groundwater flow paths and typically follow the sequence of: (1) high concentrations of dissolved oxygen, which decrease downgradient of the recharge area; (2) high concentrations of dissolved manganese and iron, indicating the active, net reduction of Mn(IV) and Fe(III) as groundwater becomes anoxic; (3) low concentrations of dissolved manganese and iron as their oxides become depleted from aquifer solids and sulfate becomes the principal electron acceptor; and (4) low concentrations of sulfate and the predominance of methanogenesis (Champ et al., 1979; Chapelle & Lovley, 1992; McMahon & Chapelle, 2008). As(V), Cr(VI), and Sb(V) also can be reduced and mobilized under the reducing conditions of zones (2), (3), and (4). At sites heavily contaminated with organics, this sequence is often reversed, with the most highly reduced zone adjacent to the source (Vroblesky & Chapelle, 1994). "Reducing conditions" is a convenient label for these situations, but it should be remembered that reactions between electron acceptors (oxidized species) and electron donors (reduced species), not "conditions", drive the observed mobilization.

D) Radioactive decay. Impacts of U, Th, Ra, Rn, and Po on drinking water derive from their radioactive decay, particularly their decay by emission of alpha particles. These radionuclides are released because of weathering reactions and mobilized by reactions with Ca^{+2} and CO_3^{-2} for U and dissolved salts for Ra (Vengosh et al., 2022). Radon derived from mineral weathering is released when water contacts the atmosphere. Polonium, which has been reported both regionally (Seiler et al., 2011) and nationally

(Szabo et al., 2020) is produced by alpha decay of Rn and likely occurs primarily in the +4 and +2 formal oxidation states (Ram et al., 2019; Szabo et al., 2020).

E) Salinization. Brackish and saline groundwater have dissolved salts and specific constituents present at concentrations that are too high for beneficial use (McMahon et al., 2016). Concentrations of the dominant, naturally occurring salts in fresh water can increase owing to evapotranspiration and natural weathering processes, including aluminosilicate weathering (panel B), evaporite dissolution, and sulfide weathering. Concentrations of naturally occurring ions can also be increased, and additional solutes that affect beneficial uses introduced, by anthropogenic processes such as application of mined nitrate fertilizers, which can have elevated concentrations of atmospherically generated perchlorate, $ClO_4^{-,}$ sea-water intrusion, and leaks or spills of oil-and-gas produced water (Böhlke, 2002; Cozzarelli et al., 2021; Hatzinger et al., 2018; McMahon et al., 2016). These processes are especially important in arid regions. Ammonium, which can threaten aquatic ecosystems by driving eutrophication and yielding toxic ammonia, is produced naturally through organic matter degradation under anaerobic conditions (Hinkle et al., 2007) and anthropogenically through releases of produced water (Farag et al., 2022; Harkness et al., 2015) and other contaminant mixtures.

F) Pipe corrosion is promoted at mildly acidic conditions, especially where high Cl/SO_4^{-2} ratios and low calcite saturation indices prevail (Jurgens et al., 2019). Dissolved oxygen (O_2), chlorine, and, for some metals (like Zn), water act as electron acceptors in the corrosion process, mobilizing metals like Zn and Cu. Lead(IV) oxide forms in pipes as a result of oxidation of elemental Pb and Pb(II) in scale by chlorine (Lytle & Schock, 2005; Stets et al., 2018). Changes in conditions brought about by changes in the source of water that flows through pipes can lead to reduction of Pb(IV) oxide to Pb^{+2}, which can be mobilized by dissolved naturally occurring organic compounds (Zhi Shi & Alan T. Stone, 2009; Z. Shi & A. T. Stone, 2009; Soeder & Miller, 2003).

Figure S2. Illustration of the study basin ranking process, each row presents 1 of the 12 variables. For each variable row, panel (a) shows the original national data set used for the variable; panel (b) shows the variable value assigned to each candidate basin; panel (c) shows the national percentile rank of each basin according to the single variable. Variables are described in the supplemental text, in Table 3, and in a data release (Qi et al., 2023).

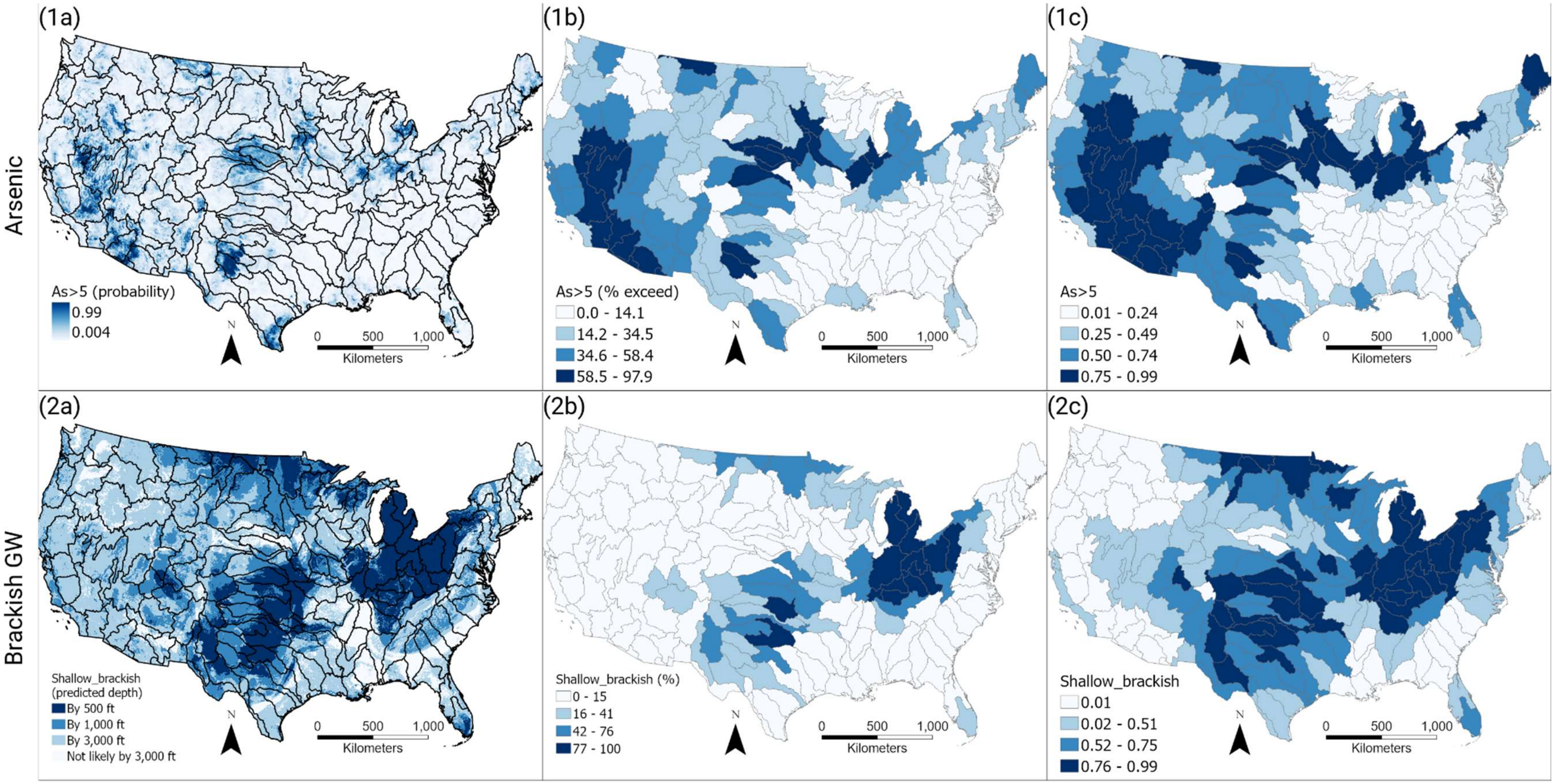

Figure S2. (continued) Illustration of the study basin ranking process, each row presents 1 of the 12 variables. For each variable row, panel (a) shows the original national data set used for the variable; panel (b) shows the variable value assigned to each candidate basin; panel (c) shows the national percentile rank of each basin according to the single variable. Variables are described in the supplemental text, in Table 3, and in a data release (Qi et al., 2023).

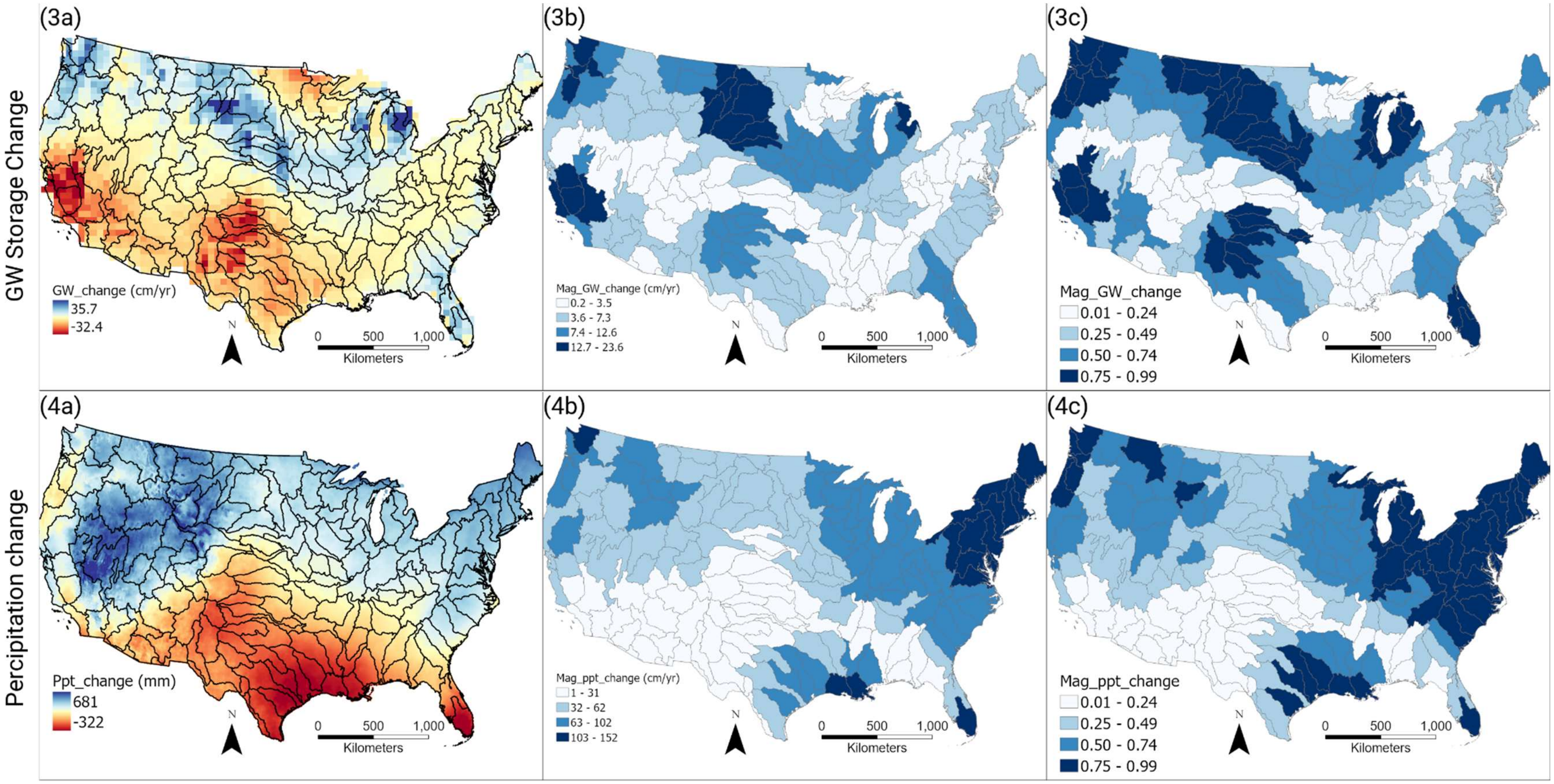

Figure S2. (continued) Illustration of the study basin ranking process, each row presents 1 of the 12 variables. For each variable row, panel (a) shows the original national data set used for the variable; panel (b) shows the variable value assigned to each candidate basin; panel (c) shows the national percentile rank of each basin according to the single variable. Variables are described in the supplemental text, in Table 3, and in a data release (Qi et al., 2023).

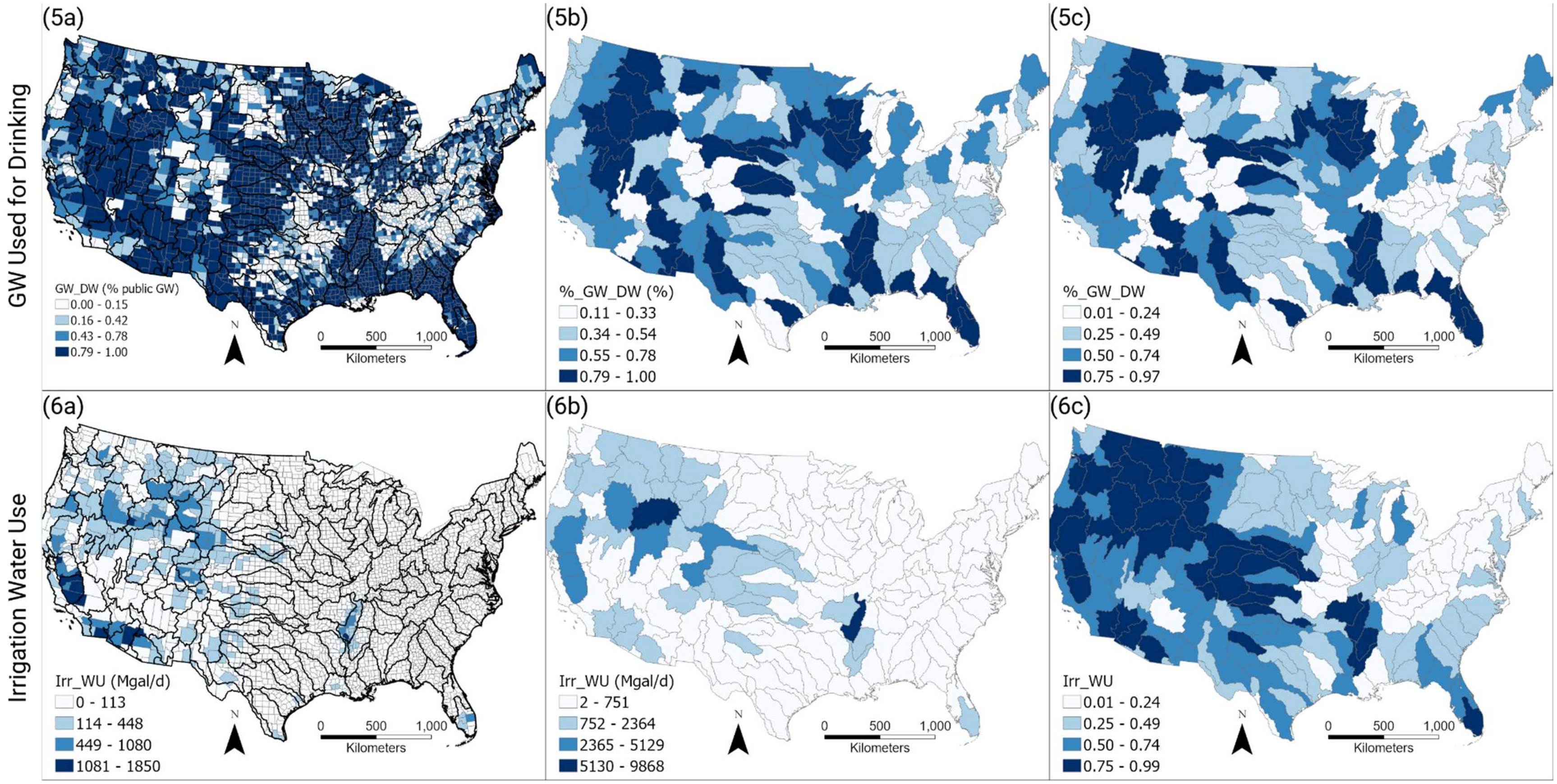

Figure S2. (continued) Illustration of the study basin ranking process, each row presents 1 of the 12 variables. For each variable row, panel (a) shows the original national data set used for the variable; panel (b) shows the variable value assigned to each candidate basin; panel (c) shows the national percentile rank of each basin according to the single variable. Variables are described in the supplemental text, in Table 3, and in a data release (Qi et al., 2023).

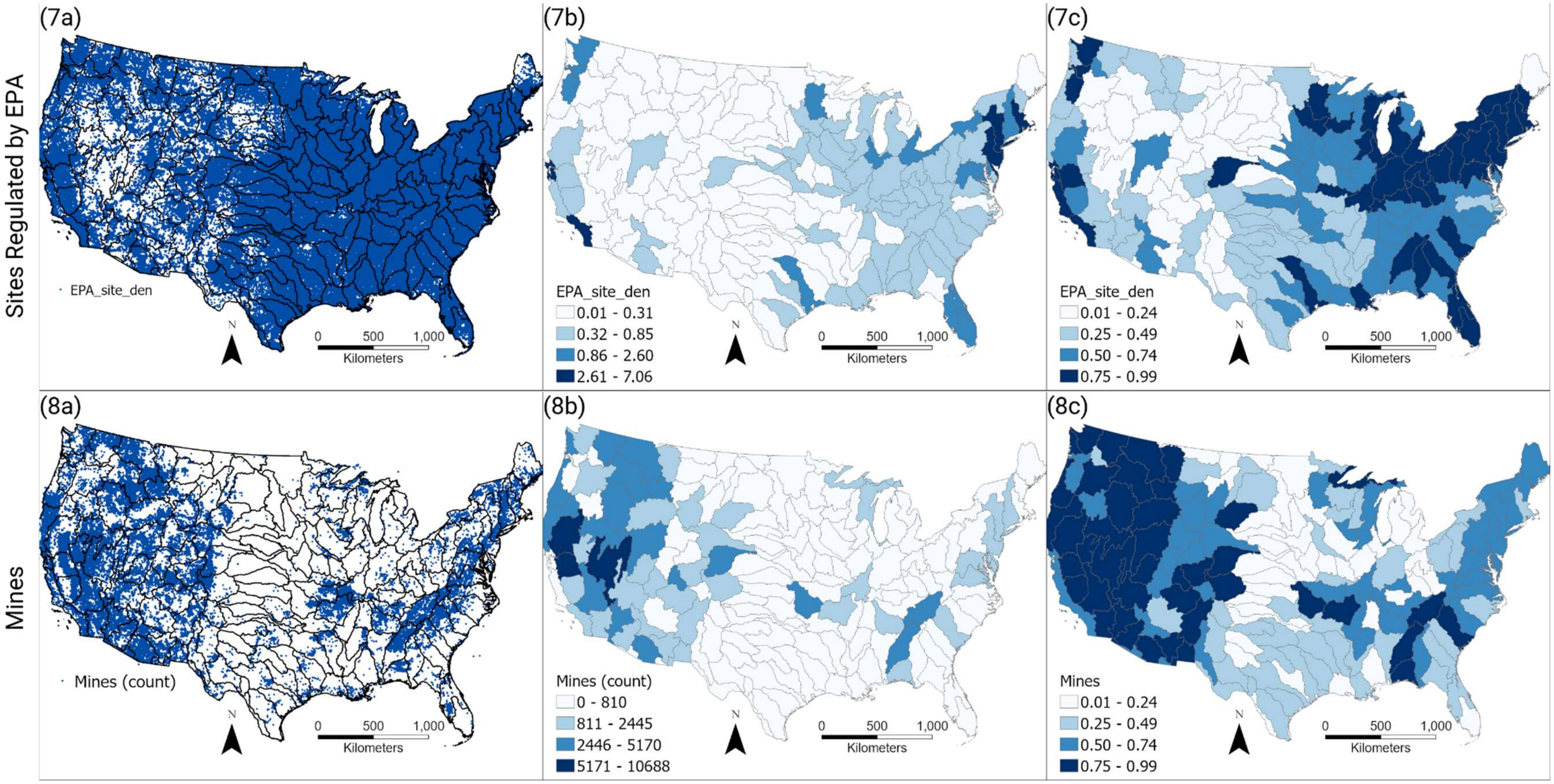

Figure S2. (continued) Illustration of the study basin ranking process, each row presents 1 of the 12 variables. For each variable row, panel (a) shows the original national data set used for the variable; panel (b) shows the variable value assigned to each candidate basin; panel (c) shows the national percentile rank of each basin according to the single variable. Variables are described in the supplemental text, in Table 3, and in a data release (Qi et al., 2023).

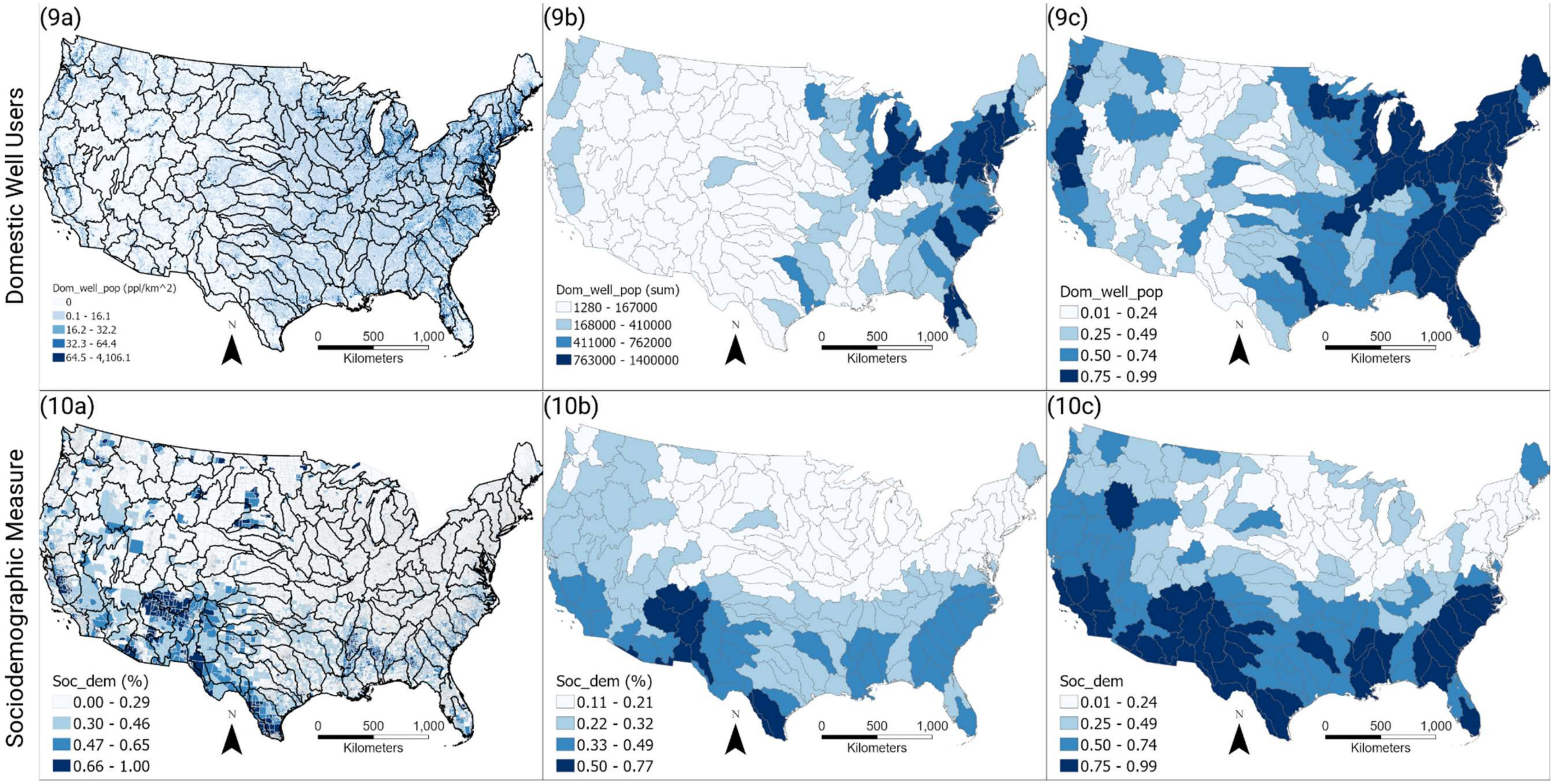

Figure S2. (continued) Illustration of the study basin ranking process, each row presents 1 of the 12 variables. For each variable row, panel (a) shows the original national data set used for the variable; panel (b) shows the variable value assigned to each candidate basin; panel (c) shows the national percentile rank of each basin according to the single variable. Variables are described in the supplemental text, in Table 3, and in a data release (Qi et al., 2023).

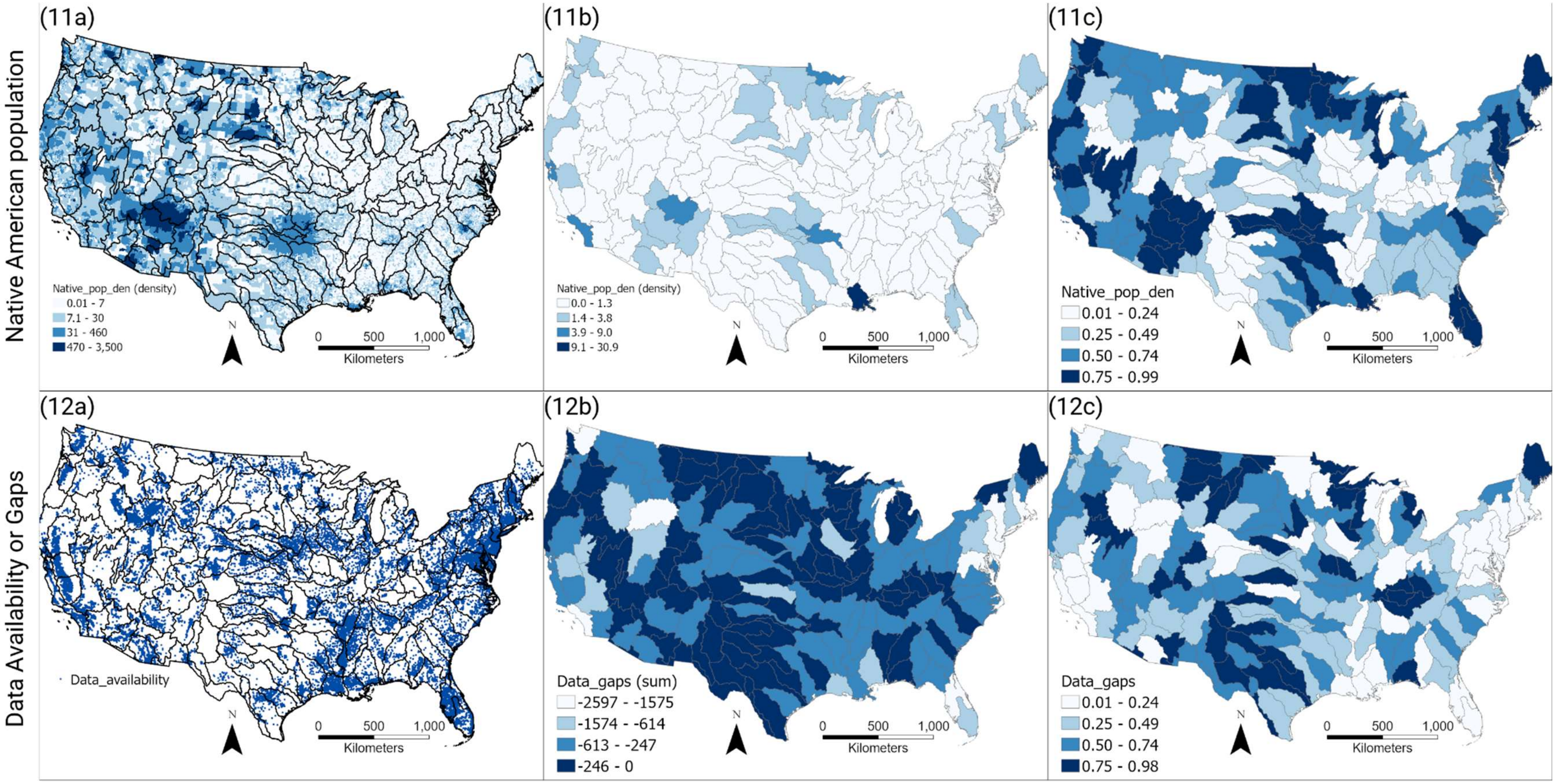

Figure S3. Map of national candidate basin ranking with selected principal aquifers (U.S. Geological Survey, 2000) for context of groundwater-source drinking water supply. Aquifer lithology can influence groundwater chemical conditions (Belitz et al., 2022).

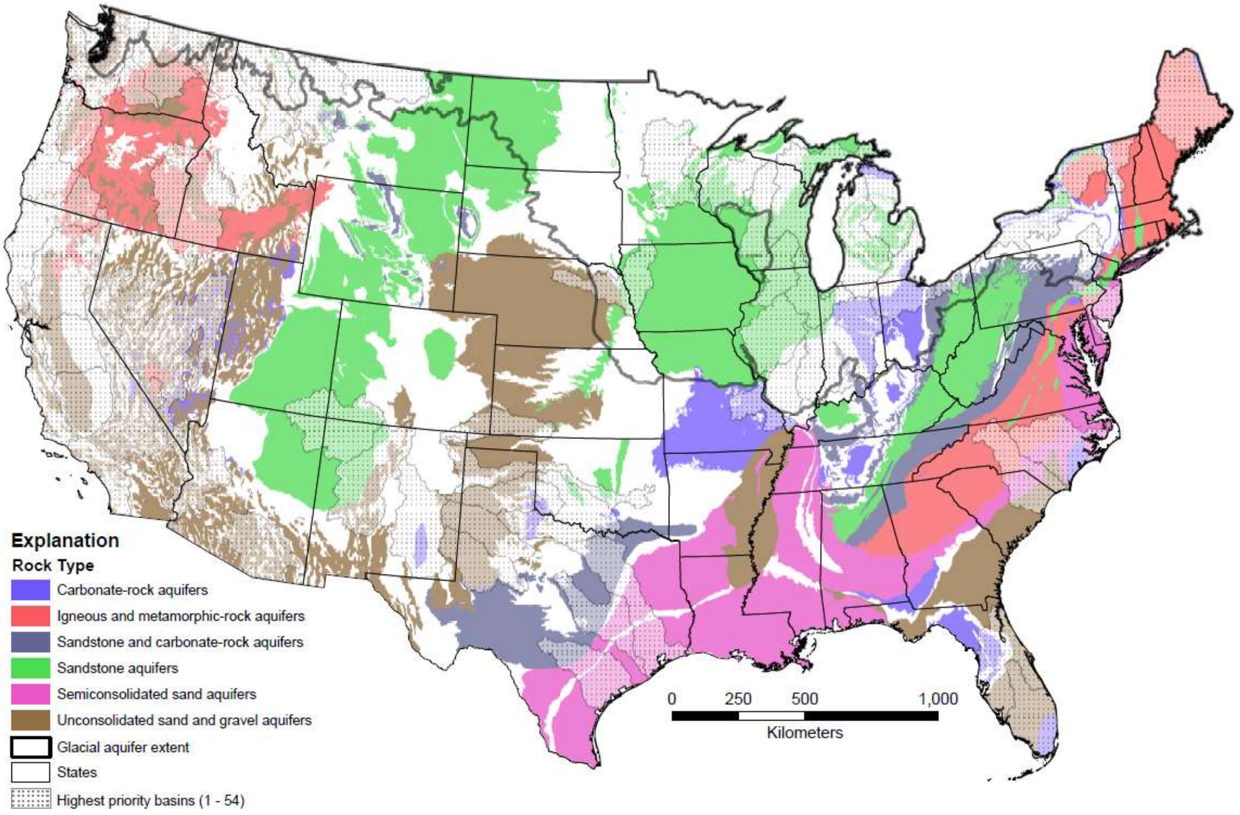

Figure S4. Percentile ranks of variable data, by region. Gray shading illustrates higher proportion of the region's data. Purple triangles indicate 3 candidate basins per region with highest regional rank, Figure S5.

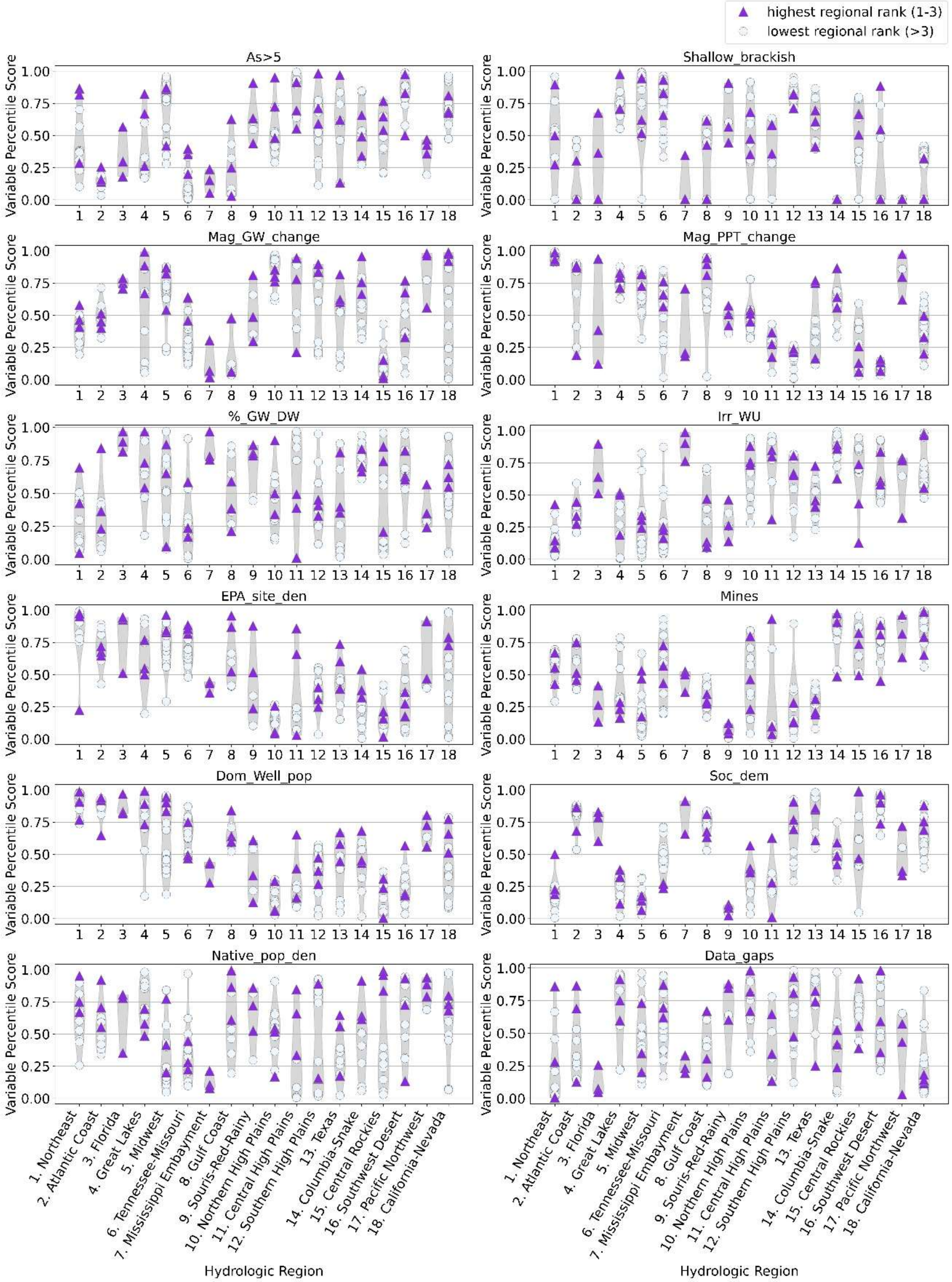

Figure S5. Maps of regional basin ranking and current (2023) selected integrated water science basins. (A) Map of regional candidate basin ranking using all variable categories. Regional ranks were assigned region by region, using candidate basins within each individual Region. Each region's candidate basin's ranks ranged from 1 to number of candidate basins in the region. (B) Selected Integrated Water Science (IWS) basins (labeled watersheds outlined in gray. To date (2023), five basins (starred) have been selected for intensive study: the Delaware River Basin, the headwaters of the Colorado and Gunnison River Basin (Upper Colorado River Basin), the Illinois River Basin, the Willamette River Basin, and the Trinity-San Jacinto River Basin (source: https://www.usgs.gov/mission-areas/water-resources/science/integrated-water-science-iws-basins).

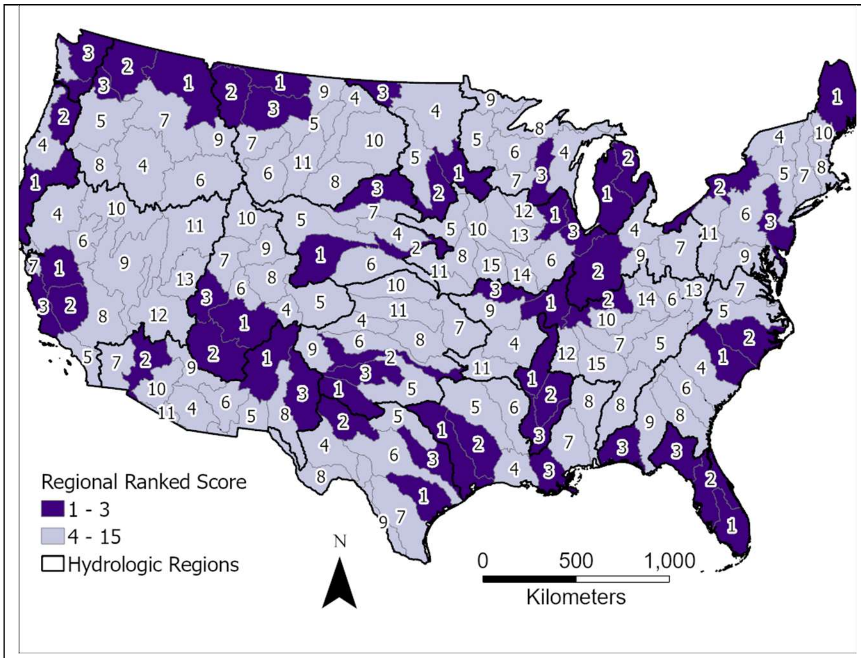

(A) Regional ranking, this study

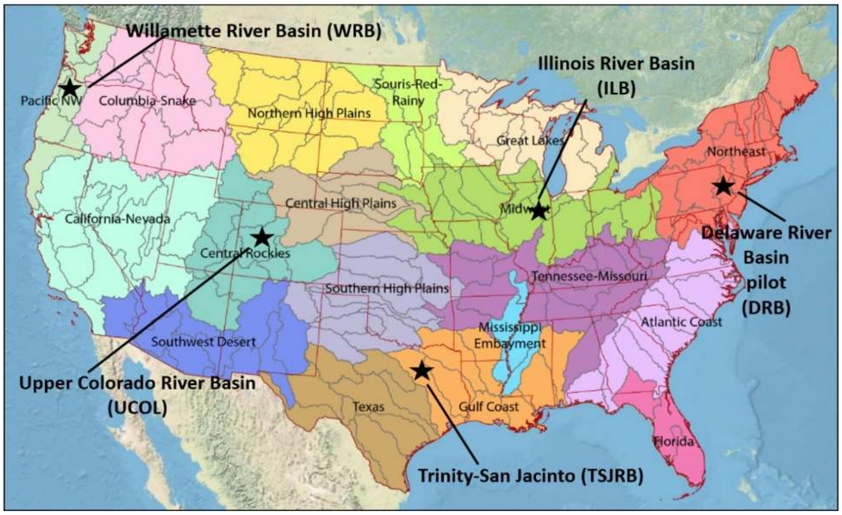

(B) Selected Integrated Water Science (IWS) basins, as of 2023 (https://www.usgs.gov/media/images/integrated-water-science-basins).
